# ﻿*Colletotrichum macroconidii* sp. nov. and six new records of *Colletotrichum* (Glomerellaceae, Glomerellales) from southwestern China

**DOI:** 10.3897/mycokeys.122.161122

**Published:** 2025-09-18

**Authors:** Chada Norphanphoun, Meng-Ting Zou, Feng-Quan Liu, Yong Wang

**Affiliations:** 1 Department of Plant Pathology, Agricultural College, Guizhou University, Guiyang, Guizhou 550025, China Guizhou University Guizhou China

**Keywords:** 1 new species, Ascomycota, molecular phylogeny, morphology, new record, species complex, taxonomy

## Abstract

*Colletotrichum*, a genus in the phylum Ascomycota and the family Glomerellaceae, is globally recognized as a significant plant pathogen affecting various hosts. In this study, 31 *Colletotrichum* strains were isolated from plant hosts in southwestern China, specifically from Guizhou and Yunnan Provinces. Phylogenetic and morphological analyses revealed one novel species, *Colletotrichum macroconidii***sp. nov.**, and 11 previously known species. Six of these species represent new host and regional records: *C. fioriniae*, *C. trichellum*, *C. juglandicola*, *C. nanhuaense*, *C. jiangxiense*, and *C. magnum*, in addition to five known species: *C. nymphaeae*, *C. metake*, *C. fructicola*, *C. siamense*, and *C. gloeosporioides*. Multilocus phylogenetic analyses placed strains of *C. jiangxiense* in a well-supported clade with *C. nullisetosum*, *C. oblongisporum*, *C. gracile*, and *C. tengchongense*, which are here treated as synonyms due to their nomenclatural invalidity, morphological overlap, and phylogenetic evidence. Similarly, *C. speciosum* and *C. simulanticitri* clustered with *C. nymphaeae* and are synonymized with it based on insignificant genetic divergence and invalid nomenclatural status. Species identification was conducted using a comprehensive approach that combined multilocus phylogenetic analysis with detailed morphological characterization. This expanded dataset significantly contributes to our understanding of the genetic diversity and ecological distribution of *Colletotrichum* in southwestern China.

## ﻿Introduction

The genus *Colletotrichum* (Glomerellaceae, Sordariomycetidae, Sordariomycetes, Ascomycota) was first described by Corda in 1831, with *C. lineola* designated as the type species. This species was originally isolated from an unidentified branch of an Apiaceae family host plant, collected in the Czech Republic. *Colletotrichum lineola* is characterized by acervuli, fusiform, curved, hyaline conidia with acute ends, and brown, opaque, subulate setae (Corda 1831). Species of *Colletotrichum* are known for their broad host range and their ability to colonize plant tissues through various infection strategies, including intracellular hemibiotrophy, subcuticular necrotrophy, and intramural necrotrophy (Bailey et al. 1992). Most *Colletotrichum* species exhibit a hemibiotrophic lifestyle, initiating infection with a symptomless biotrophic phase, which transitions into a necrotrophic phase that leads to death host cell and decay of tissue (Perfect et al. 1999; Münch et al. 2008; Crouch and Beirn 2009; Vargas et al. 2012; Damm et al. 2014; Rajarammohan 2021; Tsushima et al. 2021). In addition to their role as plant pathogens, some *Colletotrichum* species function as endophytes or saprotrophs, demonstrating their ecological versatility (Promputtha et al. 2007; Hyde and Soytong 2008; Yan et al. 2015). Currently, the genus has 874 named species, of which 764 are currently accepted in the Species Fungorum database (https://www.speciesfungorum.org; July 28, 2025). *Colletotrichum* species are globally distributed and capable of infecting a wide range of host plants, with over 1,350 documented host associations involving more than 720 plant species across diverse families, including vegetables, legumes, cereals, and both woody and herbaceous plants (Talhinhas and Baroncelli 2021). *Colletotrichum* species are among the most economically significant plant pathogens worldwide, responsible for anthracnose and blight diseases across a wide range of hosts, including fruits, vegetables, ornamental plants, and staple crops (Dean et al. 2012; Jayawardena et al. 2016b, 2021). Characteristic symptoms of *Colletotrichum* infections include sunken, dark lesions with distinct margins on leaves, stems, and fruits, leading to wilting, tissue necrosis, fruit rot, and premature fruit drop (Prusky et al. 2000; Hyde et al. 2014; Trkulja et al. 2024). These diseases contribute to significant global agricultural losses, affecting crops such as apple, mango, banana, citrus, pepper, coffee, and strawberry (Than et al. 2008; Cannon et al. 2012; Huang et al. 2013; Lima et al. 2013; Trkulja et al. 2024).

Morphologically, *Colletotrichum* species exhibit a variety of structures, including conidiomata, conidia, setae, and appresoria though considerable variation exists across species (Crouch et al. 2009). Accurate identification has historically been challenging due to the morphological similarities among species and the presence of cryptic lineages (Vieira et al. 2020; Bhunjun et al. 2021). This has led to the increased adoption of molecular approaches, such as multilocus sequence typing (MLST), utilizing genetic markers like the internal transcribed spacer (ITS), actin (*act*), beta-tubulin (*β-tubulin*), glyceraldehyde-3-phosphate dehydrogenase (*gapdh*), and chitin synthase 1 (*chs-1*) (White et al. 1990; O’Connell et al. 2012; Bhunjun et al. 2021). These molecular tools have proven essential in distinguishing closely related species and elucidating complex phylogenetic relationships within the genus. The current classification of *Colletotrichum* comprises 15 species complexes, including the *C. acutatum*, *C. agaves*, *C. boninense*, *C. caudatum*, *C. dematium*, *C. destructivum*, *C. dracaenophilum*, *C. gigasporum*, *C. gloeosporioides*, *C. graminicola*, *C. magnum*, *C. orbiculare*, *C. orchidearum*, *C. spaethianum*, and *C. truncatum* species complexes, along with numerous singletons (Marin-Felix et al. 2017; Damm et al. 2019; Jayawardena et al. 2020; Bhunjun et al. 2021). The *C. gloeosporioides* species complex is particularly notable within the genus due to its significance as a plant pathogen, with many of its members confirmed as pathogenic (Weir et al. 2012). Notably, *C. gloeosporioides* and *C. siamense* have been identified as pathogens affecting apple trees (Chen et al. 2022), rubber (Cao et al. 2019; Athinuwat et al. 2024), mango (de Souza et al. 2013), *Toxicodendron radicans* (Kasson et al. 2014), *Parthenocissus tricuspidata* (Zhao et al. 2020; Wang et al. 2025), and *Kadsura coccinea* (Jiang et al. 2022), among others.

Extensive surveys in tropical and subtropical regions have led to the discovery of numerous novel *Colletotrichum* species and newly recognized host associations, underscoring the ecological and economic importance of this genus (Armand et al. 2023b; Jiang et al. 2025; Wang et al. 2025). In China, research on *Colletotrichum* has gained significant attention due to the diverse agroecological landscapes and rich plant biodiversity (Tao et al. 2013; Liu et al. 2015, 2020, 2022, 2024; Zhang et al. 2020, 2024; Jiang et al. 2025; Lu et al. 2025; Usman et al. 2025). For example, *Colletotrichum asianum* is a pathogen causing mango anthracnose (*Mangifera* sp.), severe losses in both cultivation and postharvest stages, with yield reductions of 30%–60% (Kamle and Kumar 2016; Li et al. 2019; Wu et al. 2022). Additionally, *Colletotrichum gloeosporioides* has been identified as a pathogen causing kiwifruit anthracnose (*Actinidia chinensis*) in China, where approximately 20% of surveyed trees exhibited characteristic disease symptoms (Li et al. 2017). Major crops such as mango, tea, pepper, chili, rubber, and soybean are particularly vulnerable, with outbreaks often leading to substantial yield losses (Tao et al. 2013; Liu et al. 2015, 2022, 2024; Zhang et al. 2020, 2024; Jiang et al. 2025; Lu et al. 2025; Usman et al. 2025). Despite the challenges posed by complex pathogenicity, broad host ranges, and overlapping morphological features, recent advancements in molecular phylogenetics have greatly enhanced the resolution of *Colletotrichum* taxonomy, enabling more precise species identification and the discovery of cryptic lineages (Cai et al. 2009; Cannon et al. 2012; Marin-Felix et al. 2017; Damm et al. 2019; Jayawardena et al. 2020; Liu et al. 2022). These advancements have facilitated the development of targeted disease management strategies and underscored the importance of ongoing monitoring for effective crop protection.

This study aims to investigate *Colletotrichum* strains collected from southwestern China, with a focus on their phylogenetic diversity and morphological characteristics. By integrating molecular and morphological data, this research seeks to enhance the understanding of *Colletotrichum* diversity in the region and contribute to more accurate fungal diagnosis and management strategies in these vital ecosystems.

## ﻿Materials and methods

### ﻿Sampling and examination of specimens

The samples were collected from 2023 to 2024 in Guizhou and Yunnan Provinces, China. The specimens were taken to the laboratory in paper bags to be examined and described. Morphological characters such as conidiophore, conidiogenous cell, and conidia, were studied using a Zeiss microscope (Jena, Germany) and photographed with an AxioCam 208 color camera (Carl Zeiss Microscopy GmbH, Jena, Germany), while the size measurements were taken with the assistance of ZEN 3.0 (Blue Edition) software (Jena, Germany). Photoplates were made using Adobe Photoshop 2025 version 26.5 (Adobe Systems, CA, USA). Only the new species and newly recorded taxa are illustrated in this study.

The cultures were acquired by the tissue isolation technique as described by Norphanphoun and Hyde (2023). Single hyphal tips were transferred onto 2% Potato Dextrose Agar (PDA), Oatmeal agar (OA) and Water agar (WA) plates and incubated at room temperature (25 °C ± 2 °C): 12 hours dark and 12 hours light. The cultural features were observed and documented at 5, 7, and 14 days. Dried cultures were prepared as described by Sinclair and Dhingra (1995) and deposited at the Herbarium of the Department of Plant Pathology, Agricultural College, Guizhou University (HGUP). Dried and living cultures have been deposited in the culture collection at the Plant Pathology Department of the College of Agriculture, Guizhou University, China (**GUCC**). The enumeration for the new taxon was conducted in the MycoBank online database (https://www.mycobank.org; Robert et al. 2013).

### ﻿DNA extraction, amplification via PCR, and sequencing

Genomic DNA was extracted from fresh fungal mycelia growing on PDA at room temperature (25 °C ± 2) for two weeks using the Biospin Fungal Genomic DNA Extraction Kit (BioFlux) following the manufacturer’s protocols. Polymerase chain reactions (PCR) were carried out using the following primer pairs: ITS5/ITS4 to amplify the internal transcribed spacer region (ITS), ACT512F/ACT738R for actin (*act*), GDF/GDR for partial glyceraldehyde-3-phosphate dehydrogenase region (*gapdh*), T1/ Btub4Rd or Bt2a/Bt2b for beta-tubulin (*β-tubulin*), CHS-79F/CHS-354R for chitin synthase (*chs-1*), H3F/H3R for imidazoleglycerol-phosphate dehydratase (*his3*), and CL1C/CL2C for calmodulin (*cal*) (Weir et al. 2012; Schena et al. 2014; Kim et al. 2021; Li et al. 2021).

The amplification reactions were carried out using the following protocol: 20 μL reaction volume containing 1 µl of DNA template, 1 µL (20 µM stock concentration) of each forward and reverse primers, 10µl of 2Mix (Vazyme Biotech Co., Ltd), and 7 µl of double-distilled water (ddH_2_O). The PCR thermal cycling program for each locus is described in Table 1. The purification and sequencing of PCR products using the amplification primers specified above were conducted at Sangon Biotech (Shanghai, China) Co., Ltd. for Sanger sequencing. After sequencing, the sequence data were uploaded on GenBank, and the relevant information is listed in Table 2.

**Table 1.** Polymerase chain reactions (PCR) thermal cycling programs for each locus.

Gene	Primers	PCR thermal cycle protocols*
ITS	ITS1/ITS4	ID 95 °C for 5 min, 35 cycles of D at 95 °C for 30 s, A at 52 °C for 30 s, E at 72 °C for 1 min, FE at 72 °C for 10 min
*actin*	ACT512F/ACT738R	ID 95 °C for 5 min, 35 cycles of D at 95 °C for 30 s, A at 60.3 °C for 30 s, E at 72 °C for 1 min, FE at 72 °C for 10 min
*gapdh*	GDF/GDR	ID 95 °C for 5 min, 35 cycles of D at 95 °C for 30 s, A at 61.9 °C for 30 s, E at 72 °C for 1 min, FE at 72 °C for 10 min
*β-tubulin*	T1/T2	ID 95 °C for 5 min, 35 cycles of D at 95 °C for 30 s, A at 55.7 °C for 30 s, E at 72 °C for 1 min, FE at 72 °C for 10 min
	2a/2b	ID 95 °C for 5 min, 35 cycles of D at 95 °C for 30 s, A at 61.3 °C for 30 s, E at 72 °C for 1 min, FE at 72 °C for 10 min
*chs-1*	CHS-79F/ CHS-354R	ID 95 °C for 5 min, 35 cycles of D at 95 °C for 30 s, A at 59.4 °C for 30 s, E at 72 °C for 1 min, FE at 72 °C for 10 min
*his3*	H3F/H3R	ID 95 °C for 5 min, 35 cycles of D at 95 °C for 30 s, A at 62.1 °C for 30 s, E at 72 °C for 1 min, FE at 72 °C for 10 min
*cal*	CL1C/CL2C	ID 95 °C for 5 min, 35 cycles of D at 95 °C for 30 s, A at 62.1 °C for 30 s, E at 72 °C for 1 min, FE at 72 °C for 10 min

*ID: initial denaturation; D = denaturation; A = annealing; E = elongation; FE = final extension

**Table 2.** GenBank accession numbers of the sequences used in phylogenetic analyses in this study.

Species	Strain	Host	Location	Accession numbers								References
				ITS	gapdh	chs–1	act	β-tubulin	his	cal	ApMat	
***Colletotrichum acutatum* species complex**												
*C. abscissum*	COAD 1877 ^T^	*Citrus sinensis*	Brazil	KP843126	KP843129	KP843132	KP843141	KP843135	KP843138	–	–	Crous et al. (2015)
*C. acerbum*	CBS 128530 ^T^	*Malus domestica*	New Zealand	JQ948459	JQ948790	JQ949120	JQ949780	JQ950110	JQ949450	–	–	Damm et al. (2012)
*C. acutatum*	CBS 112996 ^T^	*Carica papaya*	Australia	JQ005776	JQ948677	JQ005797	JQ005839	JQ005860	JQ005818	–	–	Damm et al. (2012)
*C. americanum*	RGM 3380 ^T^	*Drimys winteri*	Chile	OR644563	OR644970	OR645022	OR645076	OR645128	OR659700	–	–	Zapata et al. (2024)
*C. americanum*	RGM 3129	*Laurelia sempervirens*	Chile	OR644556	OR644963	OR645015	OR645069	OR645121	OR659693	–	–	Zapata et al. (2024)
*C. arboricola*	CBS 144795 ^T^	*Fuchsia magellanica*	Chile	MH817944	MH817950	–	MH817956	MH817962	OR659702	–	–	Crous et al. (2018)
*C. australe*	CBS 116478 ^T^	*Trachycarpus fortunei*	South Africa	JQ948455	JQ948786	JQ949116	JQ949776	JQ950106	JQ949446	–	–	Damm et al. (2012)
*C. bannaense*	CGMCC 3.18887 ^T^	Rubber, leaf	China	MG209637	MG242005	MG241995	MG242001	MG209659	–	–	–	Liu et al. (2021)
*C. brisbanense*	CBS 292.67 ^T^	*Capsicum annuum*	Australia	JQ948291	JQ948621	JQ948952	JQ949612	JQ949942	JQ949282	–	–	Damm et al. (2012)
*C. cairnsense*	BRIP 63642 ^T^	*Capsicum annuum*	Australia	KU923672	KU923704	KU923710	KU923716	KU923688	KU923722	–	–	De Silva et al. (2017)
*C. carthami*	SAPA100011 ^T^	*Carthamus tinctorius*	Japan	AB696998	–	–	–	AB696992	–	–	–	Uematsu et al. (2012)
*C. chrysalidocarpi*	ZHKUCC 23-0848 ^T^	*Chrysalidocarpus lutescens*	China	OR287501	OR493925	OR493908	OR493891	OR453377	–	–	–	Zhang et al. (2023)
*C. chrysalidocarpi*	ZHKUCC 23-0847	*Chrysalidocarpus lutescens*	China	OR287500	OR493924	OR493907	OR493890	OR453376	–	–	–	Zhang et al. (2023)
*C. chrysanthemi*	IMI 364540 ^T^	*Chrysanthemum coronarium*	China	JQ948273	JQ948603	JQ948934	JQ949594	JQ949924	JQ949264	–	–	Damm et al. (2012)
*C. clavatum*	IMI 398854 ^T^	*Olea europaea*	Italy	JN121126	–	–	–	JN121213	–	–	–	Faedda et al. (2011)
*C. cosmi*	CBS 853.73 ^T^	*Cosmos* sp.	Netherlands	JQ948274	JQ948604	JQ948935	JQ949595	JQ949925	JQ949265	–	–	Damm et al. (2012)
*C. costaricense*	CBS 330.75 ^T^	*Coffea arabica*	Costa Rica	JQ948180	JQ948510	JQ948841	JQ949501	JQ949831	JQ949171	–	–	Damm et al. (2012)
*C. cuscutae*	IMI 304802 ^T^	*Cuscuta* sp.	Dominica	JQ948195	JQ948525	JQ948856	JQ949516	JQ949846	–	–	–	Damm et al. (2012)
*C. eriobotryae*	GLMC 1935 ^T^	*Eriobotrya japonica*	China	MF772487	MF795423	MN191653	MN191648	MF795428	MN191658	–	–	Damm et al. (2020)
*C. eriobotryae*	GLMC 1936	*Eriobotrya japonica*	China	MF772488	MF795424	MN191654	MN191649	MF795429	MN191659	–	–	Damm et al. (2020)
*C. filicis*	CBS 101611	Fern	Costa Rica	JQ948196	JQ948526	JQ948857	JQ949517	–	–	–	–	Damm et al. (2012)
***C. fioriniae***	**GUCC 25-0050**	***Parthenocissus tricuspidata***	**China**	**PV768508**	**PV979835**	**PV979804**	**PV979756**	**PV979905**	**PV979867**	**PV979784**	–	**In this sudy**
*C. fioriniae*	CBS 128517 ^T^	*Fiorinia externa*	USA	JQ948292	JQ948622	JQ948953	JQ949613	JQ949943	JQ949283	–	–	Damm et al. (2012)
*C. fioriniae*	CBS 129948	*Tulipa* sp.	UK	JQ948344	JQ948674	JQ949005	JQ949665	JQ949995	JQ949335	–	–	Damm et al. (2012)
*C. fioriniae*	CBS 119293	*Vaccinium corymbosum*	New Zealand	JQ948314	JQ948644	JQ948975	JQ949635	JQ949965	JQ949305	–	–	Damm et al. (2012)
*C. fioriniae*	IMI 363003, CPC 18928	*Camellia reticulata*	China	JQ948339	JQ948669	JQ949000	JQ949660	JQ949990	JQ949330	–	–	Damm et al. (2012)
*C. godetiae*	CBS 133.44 ^T^	*Clarkia hybrida*	Denmark	JQ948402	JQ948733	JQ949063	JQ949723	JQ950053	JQ949393	–	–	Damm et al. (2012)
*C. guajavae*	IMI 350839 ^T^	*Psidium guajava*	India	JQ948270	JQ948600	JQ948931	JQ949591	JQ949921	JQ949261	–	–	Damm et al. (2012)
*C. indonesiense*	CBS 127551 ^T^	*Eucalyptus* sp.	Indonesia	JQ948288	JQ948618	JQ948949	JQ949609	JQ949939	JQ949279	–	–	Damm et al. (2012)
*C. javanense*	CBS 144963a ^T^	*Capsicum annuum*	Indonesia	MH846576	MH846572	MH846573	MH846575	MH846574	MH846571	–	–	de Silva et al. (2019)
*C. johnstonii*	CBS 128532 ^T^	*Solanum lycopersicum*	New Zealand	JQ948444	JQ948775	JQ949105	JQ949765	JQ950095	JQ949435	–	–	Damm et al. (2012)
*C. kinghornii*	CBS 198.35 ^T^	*Phormium* sp.	UK	JQ948454	JQ948785	JQ949115	JQ949775	JQ950105	JQ949445	–	–	Damm et al. (2012)
*C. kniphofiae*	CBS 143496 ^T^	*Kniphofia uvaria*	UK	MH107884	MH107998	MH107990	MH107975	MH108037	–	–	–	Crous et al. (2018)
*C. laticiphilum*	CBS 112989 ^T^	*Hevea brasiliensis*	India	JQ948289	JQ948619	JQ948950	JQ949610	JQ949940	JQ949280	–	–	Damm et al. (2012)
*C. limetticola*	CBS 114.14 ^T^	*Citrus aurantiifolia*	USA, Florida	JQ948193	JQ948523	JQ948854	JQ949514	JQ949844	JQ949184	–	–	Damm et al. (2012)
*C. lupini*	CBS 109225 ^T^	*Lupinus albus*	Ukraine	JQ948155	JQ948485	JQ948816	JQ949476	JQ949806	–	–	–	Damm et al. (2012)
*C. melonis*	CBS 159.84 ^T^	*Cucumis melo*	Brazil	JQ948194	JQ948524	JQ948855	JQ949515	JQ949845	JQ949185	–	–	Damm et al. (2012)
*C. miaoliense*	NTUCC 20-001-1 ^T^	Fragaria × ananassa	China	MK908419	MK908470	MK908522	MK908573	MK908624	–	–	–	Chung et al. (2020)
*C. nagasakiense*	NBRC 116456 ^T^	*Petroselinum crispum*	Japan	LC718411	LC722608	LC722651	LC722733	LC722773	LC722691	–	–	Takata et al. (2024)
***C. nymphaeae***	**GUCC 25-0047**	**Rotten wood**	**China**	**PV768507**	**PV979834**	**PV979803**	**PV979755**	**PV979904**	**PV979866**	–	–	**In this sudy**
***C. nymphaeae***	**GUCC 25-0056**	**Rotten wood**	**China**	**PV768509**	**PV979836**	**PV979805**	**PV979757**	**PV979906**	**PX213640**	–	–	**In this sudy**
*C. nymphaeae*	CBS 515.78 ^T^	*Nymphaea alba*	Netherlands	JQ948197	JQ948527	JQ948858	JQ949518	JQ949848	JQ949188	–	–	Damm et al. (2012)
*C. nymphaeae*	CBS 134234	*Citrus aurantiifolia*	China	KC293582	KC293742	KY856139	KY855974	KC293662	KY856310	–	–	Guarnaccia et al. (2017)
*C. nymphaeae*	CBS 516.78	*Nuphar luteum*, leaf spot	Netherlands	JQ948198	JQ948528	JQ948859	JQ949519	JQ949849	JQ949189	–	–	Damm et al. (2012)
*C. nymphaeae*	CGMCC 3.15228	Nuphar lutea subsp. polysepala	USA, Florida	KC293581	KC293741	KY856138	KY855973	KC293661	KY856309	–	–	Liu et al. (2013)
*C. nymphaeae* (=*C. speciosum*)	YMF 1.07301 ^T^	*Ageratina adenophora*	China	OK030881	–	–	–	–	–	–	–	Sui et al (2024)
*C. nymphaeae* (≡*C. simulanticitri*)	YMF 1.07302	*Betula* spp.	China	OK030878	OK513680	OK513577	OK513615	–	–	–	–	Yu et al. (2022)
*C. paranaense*	CBS 134729 ^T^	*Malus domestica*	Brazil	KC204992	KC205026	KC205043	KC205077	KC205060	KC205004	–	–	Braganca et al. (2016)
*C. paxtonii*	IMI 165753 ^T^	*Musa* sp.	Saint Lucia	JQ948285	JQ948615	JQ948946	JQ949606	JQ949936	JQ949276	–	–	Damm et al. (2012)
*C. perseicola*	RGM 3376 ^T^	*Persea lingue*	Chile	OR644585	OR644992	OR645045	OR645098	OR645150	OR659723	–	–	Zapata et al. (2024)
*C. phormii*	CBS 118194 ^T^	*Phormium* sp.	Germany	JQ948446	JQ948777	JQ949107	JQ949767	JQ950097	JQ949437	–	–	Damm et al. (2012)
*C. pyricola*	CBS 128531 ^T^	*Pyrus communis*	New Zealand	JQ948445	JQ948776	JQ949106	JQ949766	JQ950096	JQ949436	–	–	Damm et al. (2012)
*C. rhombiforme*	CBS 129953 ^T^	*Olea europaea*	Portugal	JQ948457	JQ948788	JQ949118	JQ949778	JQ950108	JQ949448	–	–	Damm et al. (2012)
*C. roseum*	CBS 145754 ^T^	*Lapageria rosea*	Chile	MK903611	MK903603	–	MK903604	MK903607	OR659735	–	–	Crous et al. (2019)
*C. salicis*	CBS 607.94 ^T^	*Salix* sp.	Netherlands	JQ948460	JQ948791	JQ949121	JQ949781	JQ950111	JQ949451	–	–	Damm et al. (2012)
*C. schimae*	LC13880 ^T^	*Schima* sp.	China	MZ595885	MZ664105	MZ799347	MZ664183	MZ674003	MZ673905	–	–	Liu et al. (2022)
*C. schimae*	LC13881	*Schima* sp.	China	MZ595887	MZ664106	MZ799348	MZ664185	MZ674005	MZ673907	–	–	Liu et al. (2022)
*C. schimae*	PC9	*Carica papaya*	China	OQ642139	OQ723034	OQ723037	OQ723035	OQ723036	–	–	–	Direct Submission
*C. schimae*	CNUCC 324C-1-5-2	Camellia sinensis var. assamica	China	PP809328	PP832118	PP843051	PP824745	PP832196	PP832157	–	–	Sui et al (2024)
*C. schimae*	CNUCC 528-2-2	*Ilex chinensis*	China	PP809337	PP832126	PP843060	PP824754	PP832205	PP832166	–	–	Sui et al (2024)
*C. scovillei*	CBS 126529 ^T^	*Capsicum* sp.	Indonesia	JQ948267	JQ948597	JQ948928	JQ949588	JQ949918	JQ949258	–	–	Damm et al. (2012)
*C. simmondsii*	CBS 122122 ^T^	*Carica papaya*	Australia	JQ948276	JQ948606	JQ948937	JQ949597	JQ949927	JQ949267	–	–	Damm et al. (2012)
*C. sloanei*	IMI 364297 ^T^	*Theobroma cacao*	Malaysia	JQ948287	JQ948617	JQ948948	JQ949608	JQ949938	JQ949278	–	–	Damm et al. (2012)
*C. subsalicis*	LC13863 ^T^	*Populus alba*	China	MZ852849	–	MZ799346	MZ664128	MZ673953	MZ673836	–	–	Sui et al (2024)
*C. tamarilloi*	CBS 129814 ^T^	*Solanum betaceum*	Colombia	JQ948184	JQ948514	JQ948845	JQ949505	JQ949835	JQ949175	–	–	Damm et al. (2012)
*C. walleri*	CBS 125472 ^T^	*Coffea* sp.	Vietnam	JQ948275	JQ948605	JQ948936	JQ949596	JQ949926	JQ949266	–	–	Damm et al. (2012)
*C. wanningense*	CGMCC 3.18936 ^T^	Rubber tree	China	MG830462	MG830318	MG830302	MG830270	MG830286	–	–	–	Sui et al (2024)
*C. trichellum* (outgroup)	CBS 217.64 ^T^	*Hedera helix*	UK	GU227812	GU228204	GU228302	GU227910	GU228106	–	–	–	Damm et al. (2009)
*C. rusci* (outgroup)	CBS 119206 ^T^	*Ruscus* sp.	Italy	GU227818	GU228210	GU228308	GU227916	GU228112	GU228014	–	–	Damm et al. (2009)
***Colletotrichum bambusicola*, *coccodes*, *trichellum* species complexes**												
*C. bambusicola*	CNUCC 307307 ^T^	*Phyllostachys edulis*	China	MT199632	MT192844	MT192871	MT188638	MT192817	–	–	–	Wang et al. (2021)
*C. coccodes*	CBS 369.75 ^T^	*Solanum tuberosum*	The Netherlands	HM171679	HM171673	JQ005796	HM171667	JQ005859	JX546779	–	–	Liu et al. (2011)
*C. guangxiense*	CNUCC 310138 ^T^	*Phyllostachys edulis*	China	MT199633	MT192834	MT192861	MT188628	MT192805	–	–	–	Wang et al. (2021)
*C. hsienjenchang*	MAFF 243051 ^T^	*Phyllostachys bambusoides*	Japan	AB738855	–	AB738846	AB738845	–	AB738847	–	–	Sato et al. (2012)
*C. metake*	MAFF 244029 ^T^	*Pleioblastus simonii*	Japan	AB738859	–	–	–	OK236390	–	–	–	Sato et al. (2012)
*C. metake*	MAFF 243970	*Pleioblastus simonii*	Japan	OK090430	–	–	–	OK236391	–	–	–	Liu et al. (2022)
*C. metake*	CNUCC 311173	*Chimonobambusa quadrangularis*	China	MT192601	MT192858	MT188625	MT188652	MT192831	–	–	–	Wang et al. (2021)
*C. metake*	MAFF 241800	*Pleioblastus simonii*	Japan	OK090431	–	OK236386	OK236388	OK236392	–	–	–	Liu et al. (2022)
***C. metake***	**GUCC 25-0038**	**Bamboo**	**China**	**PV791792**	–	**PV979808**	**PV979758**	**PV979909**	**PV979871**	**PV979785**	–	**In this study**
*C. nigrum*	CBS 169.49 ^T^	*Capsicum* sp.	Argentina	JX546838	JX546742	JX546693	JX546646	JX546885	JX546791	–	–	Liu et al. (2013)
*C. nigrum* (=*C. dianense*)	CGMCC 3.18943	*Alternanthera philoxeroides*	China	–	PP482514	PP482513	PP482512	PP482516	PP482515	–	–	Chang et al. (2024)
*C. nigrum* (=*C. dianense*)	YMF 1.04943	*Alternanthera philoxeroides*	China	OL842189	OL981284	OL981310	OL981258	–	–	–	–	Zheng et al. (2022)
*C. obovoides*	LC6085 ^T^	unidentified plant, leaf	China	MZ595838	–	MZ799345	MZ664136	MZ673959	MZ673857	–	–	Liu et al. (2022)
*C. parabambusicola*	LC13884 ^T^	Bamboo, dead culm	China	MZ595904	MZ664098	MZ799338	MZ664202	MZ674022	MZ673924	–	–	Liu et al. (2022)
*C. rusci*	CBS 119206 ^T^	*Ruscus* sp.	Italy	GU227818	GU228210	GU228308	GU227916	GU228112	GU228014	–	–	Liu et al. (2013)
*C. trichellum*	CBS 217.64 ^T^	*Hedera helix*	Germany	GU227812	GU228204	GU228302	GU227910	GU228106	–	–	–	Liu et al. (2013)
*C. trichellum*	CBS 118198	*Hedera* sp.	UK	GU227813	GU228205	GU228303	GU227911	GU228107	–	–	–	Liu et al. (2013)
***C. trichellum***	**GUCC 25-0039**	***Hedera* sp.**	**China**	**PV791793**	**PV979841**	**PV979809**	**PV979759**	**PV979910**	**PV979872**	–	–	**In this study**
*C. acutatum* (outgroup)	CBS 112996 ^T^	*Carica* sp.	Australia	JQ005776	JQ948677	JQ005797	JQ005839	JQ005860	JQ005818	–	–	Liu et al. (2013)
*C. fioriniae* (outgroup)	CBS 128517 ^T^	*Fiorinia externa*	USA	JQ948292	JQ948622	JQ948953	JQ949613	JQ949943	JQ949283	–	–	Damm et al. (2012)
***Colletotrichum gloeosporioides* species complex**												
*C. aenigma*	ICMP 18608 ^T^	*Persea americana*	Israel	JX010244	JX010044	JX009774	JX009443	JX010389	–	JX009683	KM360143	Weir et al. (2012)
*C. aeschynomenes*	ICMP 17673 ^T^	*Aeschynomene virginica*	USA	JX010176	JX009930	JX009799	JX009483	JX010392	–	JX009721	KM360145	Weir et al. (2012)
*C. alatae*	CBS 304.67 ^T^	*Dioscorea alata*	India	JX010190	JX009990	JX009837	JX009471	JX010383	–	JX009738	KC888932	Weir et al. (2012)
*C. alienum*	ICMP 12071 ^T^	*Malus domestica*	New Zealand	JX010251	JX010028	JX009882	JX009572	JX010411	–	JX009654	KM360144	Weir et al. (2012)
*C. aotearoa*	ICMP 18537 ^T^	*Coprosma* sp.	New Zealand	JX010205	JX010005	JX009853	JX009564	JX010420	–	JX009611	KC888930	Weir et al. (2012)
*C. arecicola*	CGMCC 3.19667 ^T^	*Areca catechu*	China	MK914635	MK935455	MK935541	MK935374	MK935498	–	–	MK935413	Cao et al. (2020)
*C. artocarpicola*	MFLUCC 18-1167 ^T^	* Artocarpus *	Thailand	MN415991	MN435568	MN435569	MN435570	MN435567	–	–	–	Bhunjun et al. (2019)
*C. asianum*	ICMP 18580 ^T^	*Coffea arabica*	Thailand	FJ972612	JX010053	JX009867	JX009584	JX010406	–	FJ917506	FR718814	Zhang et al. (2023)
*C. australianum*	VPRI 43075 ^T^	*Citrus sinensis*	Australia, Vic	MG572138	MG572127	MW091987	MN442109	MG572149	–	–	MG572171	Wang et a. (2021)
*C. camelliae*	CGMCC 3.14925 ^T^	*Camellia sinensis*	China	KJ955081	KJ954782		KJ954363	KJ955230	–	KJ954634	KJ954497	Wang et a. (2021)
*C. camelliae* (≡ *C. analogum*)	YMF1.06943 ^T^	*Ageratina adenophora*	China	OK030860	OK513663	OK513559	OK513599	OK513629	–	–	PP498774	Yu et al. (2022)
*C. cangyuanense*	YMF1.05001^T^	*Ageratina adenophora*	China	OK030864	OK513667	OK513563	OK513603	OK513633	–	–	–	Yu et al. (2022)
*C. castaneae*	GUCC 21268.4 ^T^	*Castanea mollissima*	China	OP722991	OP737973	OP715778	OP715812	OP720868	–	–	–	Zhang et al. (2023)
*C. changpingense*	MFLUCC 15-0022 ^T^	*Fragaria × ananassa*	China	KP683152	KP852469	KP852449	KP683093	KP852490	–	–	–	Wang et a. (2021)
*C. chiangmaiense*	MFLUCC 18-0945 ^T^	Magnolia garrettii	Thailand	MW346499	MW548592	MW623653	MW655578		–	–	–	Zhang et al. (2023)
*C. chrysophilum*	CMM4268 ^T^	*Musa* sp.	Brazil	KX094252	KX094183	KX094083	KX093982	KX094285	–	KX094063	KX094325	Wang et a. (2021)
*C. cigarro*	ICMP 18539 ^T^	*Olea europaea*	Australia	JX010230	JX009966	JX009800	JX009523	JX010434	–	JX009635	–	Weir et al. (2012)
*C. clidemiae*	ICMP 18658 ^T^	*Clidemia hirta*	USA, Hawaii	JX010265	JX009989	JX009877	JX009537	JX010438	–	JX009645	KC888929	Weir et al. (2012)
*C. cobbittiense*	BRIP 66219a ^T^	*Magnolia garrettii*	Thailand	MH087016	MH094133	MH094135	MH094134	MH094137	–	–	–	Zhang et al. (2023)
*C. conoides*	CAUG17 ^T^	*Capsicum annuum*	China	KP890168	KP890162	KP890156	KP890144	KP890174	–	KP890150	–	Wang et a. (2021)
*C. cordylinicola*	ICMP 18579 ^T^	*Cordyline fruticosa*	Thailand	JX010226	JX009975	JX009864	HM470235	JX010440	–	HM470238	JQ899274	Weir et al. (2012)
*C. cycadis*	BRIP 71326a ^T^	*Cycas revoluta*	China	MT439915	MT439919	MT439917		MT439921	–	–		Zhang et al. (2023)
*C. dimorphum*	YMF1.07309 ^T^	*Ageratina adenophora*	China	OK030867	OK513670	OK513566	OK513606	OK513636	–	–	PP498776	Yu et al. (2022)
*C. dracaenigenum*	MFLUCC 19-0430 ^T^	*Dracaena* sp.	Thailand	MN921250	MT215577	MT215575	MT313686		–	–	–	Zhang et al. (2023)
*C. endophyticum*	MFLUCC 13-0418 ^T^	*Pennisetum purpureum*	Thailand	KC633854	KC832854	MZ799261	KF306258	MZ673954	–	KC810018	–	Zhang et al. (2023)
*C. fici-septicae*	MFLUCC 20-0166 ^T^	*Ficus septica*	China	MW114367	MW183774	MW177701	MW151585		–	–	–	Zhang et al. (2023)
*C. fructicola*	MFLUCC 22-0181	Pineapple	Thailand	OQ048649	OQ067350	OQ067349	OQ067348	OQ067351	–	–	–	Armand et al. (2023a)
*C. fructicola*	MFLUCC 22-0182	Pineapple	Thailand	OQ048650	OQ067354	OQ067353	OQ067352	OQ067355	–	–	–	Armand et al. (2023a)
*C. fructicola*	MFLUCC 17-1752	*Rhizophora apiculata*	Thailand	OR828931	OR840868	OR840856	OR840845	OR840862	–	OR840851	–	Norphanphoun and Hyde (2023)
*C. fructicola*	MFLUCC 17-1753	*Rhizophora apiculata*	Thailand	OR828932	OR840869	OR840857	OR840846	OR840863	–	OR840852	–	Norphanphoun and Hyde (2023)
*C. fructicola*	ICMP 18581 ^T^	*Coffea arabica*	Thailand	JX010165	JX010033	JX009866	FJ907426	JX010405	–	FJ917508	JQ807838	Weir et al. (2012)
*C. fructicola*	ICMP 18613	*Limonium sinuatum*	Israel	JX010167	JX009998	JX009772	JX009491	JX010388	–	JX009675	–	Weir et al. (2012)
*C. fructicola*	ICMP 18727	Fragaria × ananassa	USA	JX010179	JX010035	JX009812	JX009565	JX010394	–	JX009682	–	Weir et al. (2012)
***C. fructicola***	**GUCC 25-0021**	***Juglans regia***	**China**	**PV791817**	**PV979865**	**PV979833**	**PV979783**	**PV979934**	**PV979896**	**PV979786**	**PV979935**	**In this study**
***C. fructicola***	**GUCC 25-0022**	***Juglans regia***	**China**	**PV791816**	**PV979864**	**PV979832**	**PV979782**	**PV979933**	**PV979895**	**PV979787**	**PV979936**	**In this study**
***C. fructicola***	**GUCC 25-0023**	***Juglans regia***	**China**	**PV791815**	**PV979863**	**PV979831**	**PV979781**	**PV979932**	**PV979894**	**PV979788**	**PV979937**	**In this study**
***C. fructicola***	**GUCC 25-0024**	***Juglans regia***	**China**	**PV791814**	**PV979862**	**PV979830**	**PV979780**	**PV979931**	**PV979893**	**PV979789**	**PV979938**	**In this study**
***C. fructicola***	**GUCC 25-0025**	***Juglans regia***	**China**	**PV791813**	**PV979861**	**PV979829**	**PV979779**	**PV979930**	**PV979892**	**PV979790**	**PV979939**	**In this study**
***C. fructicola***	**GUCC 25-0045**	**Actinidia chinensis var. deliciosa**	**China**	**PV791799**	**PV979847**	**PV979815**	**PV979765**	**PV979916**	**PV979878**	**PV979800**	**PV979947**	**In this study**
***C. fructicola***	**GUCC 25-0051**	***Rosa chinensis***	**China**	**PV791796**	**PV979844**	**PV979812**	**PV979762**	**PV979913**	**PV979875**	**PV979801**	–	**In this study**
*C. fructivorum*	CBS 124.22	n/a	USA	MH854714	–	–	–	JX145176	–	–	–	Vu et al. (2019)
*C. fructivorum*	CBS 133125 ^T^	*Vaccinium macrocarpon*	USA	JX145145	MZ664047	MZ799259	MZ664126	JX145196	–	–	JX145300	Wang et a. (2021)
*C. gardeniae*	GUCC 12049 ^T^	*Gardenia jasminoides*	China	OP722995	OP737963	OP715766	OP715801	OP720858	–	–	–	Zhang et al. (2023)
*C. gardeniae*	GUCC 12048	*Gardenia jasminoides*	China	OP722989	OP737962	OP715765	OP715800	OP720857	–	–	–	Zhang et al. (2023)
*C. gardeniae*	GUCC 12047	*Gardenia jasminoides*	China	OP722964	OP737961	OP715764	OP715799	OP720856	–	–	–	Zhang et al. (2023)
*C. gloeosporioides*	IMI 356878 ^T^	*Citrus sinensis*	Italy	JQ005152	JQ005239	JQ005326	JQ005500	JQ005587	–	–	–	Damm et al. (2012)
*C. gloeosporioides*	LGMF800	*Commelina benghalensis*	Rincão	KM278577	KM257052	KJ579936	KJ569194	KJ579891	–	–	–	Waculicz-Andrade et al. (2017)
*C. gloeosporioides*	LGMF748	*Sida rhombifolia*	Rincão	KM257023	KM257047	KJ579931	KJ569189	KJ579839	–	–	–	Waculicz-Andrade et al. (2017)
*C. gloeosporioides*	LGMF524	* Citrus *	Mogi Guaçú	JQ580610	JQ580791	KJ579927	KJ569185	KJ579739	–	–	–	Waculicz-Andrade et al. (2017)
*C. gloeosporioides*	ICMP 18730	*Citrus* sp.	New Zealand	JX010157	JX009981	JX009861	JX009548		–	–	–	Weir et al. (2012)
*C. gloeosporioides*	CBS 112999 ^T^	*Citrus sinensis*	Italy	JX010152	JX010056	JX009818	JX009531	JX010445	–	JX009731	JQ807843	Weir et al. (2012)
*C. gloeosporioides*	ICMP 19121	*Citrus limon*	Italy	JX010148	JX010054	JX009903	JX009558		–	–	–	Weir et al. (2012)
***C. gloeosporioides***	**GUCC 25-0041**	***Camellia Japonica***	**China**	**PV791803**	**PV979851**	**PV979819**	**PV979769**	**PV979920**	**PV979882**	–	–	**In this study**
***C. gloeosporioides***	**GUCC 25-0042**	***Camellia Japonica***	**China**	**PV791802**	**PV979850**	**PV979818**	**PV979768**	**PV979919**	**PV979881**	–	–	**In this study**
*C. grevilleae*	CBS 132879 ^T^	*Grevillea* sp.	Italy	KC297078	KC297010	KC296987	KC296941	KC297102	–	KC296963	–	Wang et a. (2021)
*C. grossi*	CAUG7 ^T^	*Capsicum* sp.	China	KP890165	KP890159	KP890153	KP890141	KP890171	–	KP890147	–	Wang et a. (2021)
*C. hebeiense*	MFLUCC 13-0726 ^T^	*Vitis vinifera* cv. *Cabernet Sauvignon*	China	KF156863	KF377495	KF289008	KF377532	KF288975	–	–	KF377562	Wang et a. (2021)
*C. hederiicola*	MFLU 15-0689 ^T^	*Hedera helix*	Italy	MN631384	–	MN635794	MN635795	–	–	–	–	Zhang et al. (2023)
*C. helleniense*	CBS 142418 ^T^	*Poncirus trifoliata*	Greece	KY856446	KY856270	KY856186	KY856019	KY856528	–	KY856099	–	Wang et a. (2021)
*C. henanense*	CGMCC 3.17354 ^T^	*Camellia sinensis*	China	KJ955109	KJ954810	–	KM023257	KJ955257	–	KJ954662	KJ954524	Wang et a. (2021)
*C. horii*	ICMP 10492 ^NT^	*Diospyros kaki*	Japan	GQ329690	GQ329681	JX009752	JX009438	JX010450	–	JX009604	JQ807840	Wang et a. (2021)
*C. hystricis*	CBS 142411 ^T^	*Citrus hystrix*	Italy	KY856450	KY856274	KY856190	KY856023	KY856532	–	KY856103	–	Wang et a. (2021)
*C. jiangxiense*	22N642	n/a	South Korea	OR805434	OR826297	OR826295	–	OR826299	–	–	–	Direct Submission
*C. jiangxiense*	SYD-9	n/a	China	OR467495	OR472539	OR472537	OR472535	OR472541	–	–	–	Direct Submission
*C. jiangxiense*	SYD-4	n/a	China	OR467494	OR472538	OR472536	OR472534	OR472540	–	–	–	Direct Submission
*C. jiangxiense*	CGMCC 3.17363 ^T^	*Camellia sinensis*	China	KJ955201	KJ954902	–	KJ954471	KJ955348	–	KJ954752	KJ954607	Liu et al. (2015)
***C. jiangxiense***	**GUCC 25-0027**	***Juglans regia***	**China**	**PV791811**	**PV979859**	**PV979827**	**PV979777**	**PV979928**	**PV979890**	**PV979792**	**PV979940**	**In this study**
***C. jiangxiense***	**GUCC 25-0031**	***Juglans regia***	**China**	**PV791808**	**PV979856**	**PV979824**	**PV979774**	**PV979925**	**PV979887**	**PV979795**	**PV979943**	**In this study**
***C. jiangxiense***	**GUCC 25-0052**	***Pteris henryi***	**China**	**PV791795**	**PV979843**	**PV979811**	**PV979761**	**PV979912**	**PV979874**	–	–	**In this study**
***C. jiangxiense***	**GUCC 25-0053**	***Parthenocissus tricuspidata***	**China**	**PV791794**	**PV979842**	**PV979810**	**PV979760**	**PV979911**	**PV979873**	**PV979802**	**PV979948**	**In this study**
*C. jiangxiense* (=*C. gracile*)	YMF 1.07329	*Ageratina adenophora*	China	OK030869	OK513672	OK513568	OK513608	OK513638	–	–	–	Yu et al. (2022)
*C. jiangxiense* (=*C. gracile*)	YMF1.06939 ^T^	*Ageratina adenophora*	China	OK030868	OK513671	OK513567	OK513607	OK513637	–	–	PP498777	Yu et al. (2022)
*C. jiangxiense* (=*C. nullisetosum*)	YMF1.06946 ^T^	Mango	China	OK030872	OK513675	OK513571	OK513611	OK513641	–	–	PP498779	Yu et al. (2022)
*C. jiangxiense* (=*C. nullisetosum*)	YMF1.07328	Mango	China	OK030873	OK513676	OK513572	OK513612	OK513642	–	–	–	Yu et al. (2022)
*C. jiangxiense* (=*C. oblongisporum*)	YMF1.06938 ^T^	*Ageratina adenophora*	China	OK030874	OK513677	OK513573	–	OK513643	–	–	PP498780	Yu et al. (2022)
*C. jiangxiense* (=*C. oblongisporum*)	YMF 1.07326	*Ageratina adenophora*	China	OK030875	OK513678	OK513574	–	OK513644	–	–	–	Yu et al. (2022)
*C. juglandicola*	CGMCC3.24312 ^T^	*Juglans regia*	China	OQ263015	OQ282973	OR004793	OQ282966	OQ282980	–	–	–	Zhang et al. (2023)
*C. juglandicola*	CGMCC3.24313	*Juglans regia*	China	OQ263018	OQ282977	OR004797	OQ282970	OQ282984	–	–	–	Zhang et al. (2023)
***C. juglandicola***	**GUCC 25-0043**	***Camellia Japonica***	**China**	**PV791801**	**PV979849**	**PV979817**	**PV979767**	**PV979918**	**PV979880**	**PV979798**	**PV979945**	**In this study**
***C. juglandicola***	**GUCC 25-0044**	***Ilex* sp.**	**China**	**PV791800**	**PV979848**	**PV979816**	**PV979766**	**PV979917**	**PV979879**	**PV979799**	**PV979946**	**In this study**
*C. kahawae*	CIFC Uga7	*Coffea arabica*	Uganda: Kapchorwa	HE655531	–	–	–	HE655592	–	–	HE657410	Silva et al. (2012)
*C. kahawae*	ICMP 17816 ^T^	*Coffea arabica*	Kenya	JX010231	JX010012	JX009813	JX009452	JX010444	–	JX009642	JQ894579	Weir et al. (2012)
*C. kahawae*	ICMP:17811	*Coffea arabica*	Malawi	JX010233	JX009970	JX009817	JX009555	JX010430	–	JX009641	OQ871550	Weir et al. (2012)
*C. kunmingense*	GUCC 12053 ^T^	*Ophiopogon japonicus*	China	OP722975	OP737965	OP715769	OP715804	OP720861	–	–	–	Zhang et al. (2023)
*C. ledongense*	CGMCC3.18888 ^T^	*Hevea brasiliensis*	China	MG242009	MG242017	MG242019	MG242015	MG242011	–	MG242013	–	Liu et al. (2018)
*C. ligustri*	GUCC 12111 ^T^	*Ilex chinensis*	China	OP722988	OP737968	OP715773	OP740216	OP720864	–	–	–	Zhang et al. (2023)
***C. macroconidii***	**GUCC 25-0028 ^T^**	***Juglans regia***	**China**	**PV791810**	**PV979858**	**PV979826**	**PV979776**	**PV979927**	**PV979888**	**PV979793**	**PV979941**	**In this study**
***C. macroconidii***	**GUCC 25-0063**	***Juglans regia***	**China**	**PV791809**	**PV979857**	**PV979825**	**PV979775**	**PV979926**	**PV979889**	**PV979794**	**PV979942**	**In this study**
*C. makassarense*	CBS 143664 ^T^	*Capsicum annuum*	Indonesia	MH728812	MH728820	MH805850	MH781480	MH846563	–	–	MH728831	Zhang et al. (2023)
*C. menglaense*	YMF1.04960 ^T^	Air	China	MH023505	MH023507	MH023508	MH023506		–	–	–	Zhang et al. (2023)
*C. musae*	CBS 116870 ^T^	*Musa* sp.	USA	JX010146	JX010050	JX009896	JX009433	HQ596280	–	JX009742	KC888926	Weir et al. (2012)
*C. nanhuaense*	YMF1.04993 ^T^	*Ageratina adenophora*	China	OK030870	OK513673	OK513569	OK513609	OK513639	–	–	PP498778	Yu et al. (2022)
***C. nanhuaense***	**GUCC 25-0026**	***Juglans regia***	**China**	**PV791812**	**PV979860**	**PV979828**	**PV979778**	**PV979929**	**PV979891**	**PV979791**	–	**In this study**
*C. nupharicola*	CBS 470.96 ^T^	Nuphar lutea subsp. polysepala	USA	JX010187	JX009972	JX009835	JX009437	JX010398	–	JX009663	JX145319	Weir et al. (2012)
*C. perseae*	CBS 141365 ^T^	*Persea americana*	Israel	KX620308	KX620242	–	KX620145	KX620341	–	KX620206	KX620177	Sharma et al. (2017)
*C. proteae*	CBS 132882 ^T^	*Protea* sp.	South Africa	KC297079	KC297009	KC296986	KC296940	KC297101	–	KC296960	–	Wang et a. (2021)
*C. pseudotheobromicola*	MFLUCC 18-1602 ^T^	*Avocado*	Israel	MH817395	MH853675	MH853678	MH853681	MH853684	–	–	–	Zhang et al. (2023)
*C. psidii*	CBS 145.29 ^T^	*Psidium* sp.	Italy	JX010219	JX009967	JX009901	JX009515	JX010443	–	JX009743	KC888931	Weir et al. (2012)
*C. queenslandicum*	ICMP 1778 ^T^	*Carica papaya*	Australia	JX010276	JX009934	JX009899	JX009447	JX010414	–	JX009691	KC888928	Weir et al. (2012)
*C. rhexiae*	CBS 133134 ^T^	*Rhexia virginica*	USA	JX145128	MZ664046	MZ799258	MZ664127	JX145179	–	–	JX145290	Zhang et al. (2023)
*C. rhizophorae*	MFLUCC 17-1927 ^T^	*Rhizophora apiculata*	Thailand	OR828933	OR840870	OR840858	OR840847	OR840864	–	OR840853	–	Norphanphoun and Hyde (2023)
*C. salsolae*	ICMP 19051 ^T^	*Salsola tragus*	Hungary	JX010242	JX009916	JX009863	JX009562	JX010403	–	JX009696	KC888925	Weir et al. (2012)
*C. siamense*	MFLUCC 18-1162	*Rosa* sp.	Thailand	MN788676	MN995328	MN995335	MN995334	MN995329	–	–	–	Hyde et al. (2020)
*C. siamense*	MFLUCC 22-0138	*Cyclosorus* sp.	Thailand	OP802366	OP801723	OP801705	OP801688	OP801742	–	–	–	Seifollahi et al. (2023)
*C. siamense*	ICMP 18578 ^T^	*Coffea arabica*	Thailand	JX010171	JX009924	JX009865	FJ907423	JX010404	–	FJ917505	JQ899289	Weir et al. (2012)
*C. siamense*	ICMP 18572	*Vitis vinifera*	USA	JX010160	JX010061	JX009783	JX009487	–	–	–	–	Weir et al. (2012)
*C. siamense*	ICMP 18739	Carica papaya	South Africa	JX010161	JX009921	JX009794	JX009484	–	–	–	–	Weir et al. (2012)
*C. siamense*	ICMP 18571	Fragaria × ananassa	USA	JX010159	JX009922	JX009782	JX009482	–	–	–	–	Weir et al. (2012)
*C. siamense*	MFLUCC 17-0571	* Pandanaceae *	Thailand	MG646967	MG646934	MG646931	MG646938	MG646926	–	–	–	Wang et a. (2021)
*C. siamense* (= *C. parvisporum*)	SAUCC 201152	n/a	China	MW786746	MW876478	MW883693	MW883702	MW888977	–	–	–	Direct Submission
*C. siamense* (= *C. parvisporum*)	SAUCC 200204	n/a	China	MW786641	MW846239	MW883685	MW883694	MW888969	–	–	–	Direct Submission
*C. siamense* (= *C. parvisporum*)	MFLUCC 22-0151	*Cyclosorus* sp., leaf	Thailand	OP802371	OP801726	OP801708	OP801691	OP801746	–	–	–	Seifollahi et al. (2023)
*C. siamense* (= *C. parvisporum*)	MFLUCC 22-0164	*Cyclosorus* sp., leaf	Thailand	OP802369	OP801724	OP801706	OP801689	OP801744	–	–	–	Seifollahi et al. (2023)
*C. siamense* (= *C. parvisporum*)	MFLUCC 22-0159	*Nephrolepis cordifolia*	Thailand	OP802373	OP801727	OP801709	OP801692	OP801747	–	–	–	Seifollahi et al. (2023)
*C. siamense* (= *C. parvisporum*)	YMF 1.06942	*Ageratina adenophora*	China	OK030876	OK513679	OK513575	OK513613	OK513645	–	–	–	Yu et al. (2022)
***C. siamense***	**GUCC 25-0032**	***Juglans regia***	**China**	**PV791807**	**PV979855**	**PV979823**	**PV979773**	**PV979924**	**PV979886**	**PV979796**	**PV979944**	**In this study**
***C. siamense***	**GUCC 25-0033**	** Pteridophyta **	**China**	**PV791806**	**PV979854**	**PV979822**	**PV979772**	**PV979923**	**PV979885**	**PV979797**	–	**In this study**
***C. siamense***	**GUCC 25-0034**	** Pteridophyta **	**China**	**PV791805**	**PV979853**	**PV979821**	**PV979771**	**PV979922**	**PV979884**	–	–	**In this study**
***C. siamense***	**GUCC 25-0035**	** Pteridophyta **	**China**	**PV791804**	**PV979852**	**PV979820**	**PV979770**	**PV979921**	**PV979883**	–	–	**In this study**
***C. siamense***	**GUCC 25-0048**	***Parthenocissus quinquefolia***	**China**	**PV791798**	**PV979846**	**PV979814**	**PV979764**	**PV979915**	**PV979877**	–	–	**In this study**
***C. siamense***	**GUCC 25-0049**	***Parthenocissus tricuspidata***	**China**	**PV791797**	**PV979845**	**PV979813**	**PV979763**	**PV979914**	**PV979876**	–	–	**In this study**
*C. subhenanense*	YMF1.06865 ^T^	*Ageratina adenophora*	China	OK030883	OK513684	OK513581	OK513618	OK513647	–	–	PP498782	Yu et al. (2022)
*C. syzygiicola*	MFLUCC 10-0624 ^T^	*Syzygium samarangense*	Thailand	KF242094	KF242156	–	KF157801	KF254880	–	KF254859	–	Wang et a. (2021)
*C. tainanense*	CBS 143666 ^T^	*Capsicum annuum*	China	MH728818	MH728823	MH805845	MH781475	MH846558	–	–	MH728836	Zhang et al. (2023)
*C. temperatum*	CBS 133122 ^T^	*Vaccinium macrocarpon*	USA	JX145159	MZ664045	MZ799254	MZ664125	JX145211	–	–	JX145298	Wang et a. (2021)
*C. tengchongense*	YMF 1.04950 ^T^	*Isoetes sinensis*	China	OL842169	OL981264	OL981290	PP498771	PP498785	–	PP498784	PP498773	Zhang et al. (2023)
*C. thailandica*	MFLUCC 17-1924 ^T^	*Rhizophora apiculata*	Thailand	OR828935	OR840872	OR840860	OR840849	OR840866	–	OR840855		Norphanphoun and Hyde (2023)
*C. theobromicola*	CBS 124945 ^T^	*Theobroma cacao*	Panama	JX010294	JX010006	JX009869	JX009444	JX010447	–	JX009591	KC790726	Weir et al. (2012)
*C. ti*	ICMP 4832 ^T^	*Cordyline* sp.	New Zealand	JX010269	JX009952	JX009898	JX009520	JX010442	–	JX009649	KM360146	Weir et al. (2012)
*C. tropicale*	CBS 124949 ^T^	*Theobroma cacao*	Panama	JX010264	JX010007	JX009870	JX009489	JX010407	–	JX009719	KC790728	Weir et al. (2012)
*C. viniferum*	GZAAS5.08601 ^T^	*Vitis vinifera*, cv. ‘*Shuijing*’	China	JN412804	JN412798	–	JN412795	JN412813	–	JQ309639		Wang et a. (2021)
*C. vulgaris*	YMF 1.04940 ^T^	*Hippuris vulgaris*	China	OL842170	OL981265	OL981291	OL981239	–	–			Zhang et al. (2023)
*C. wuxiense*	CGMCC 3.17894 ^T^	*Camellia sinensis*	China	KU251591	KU252045	KU251939	KU251672	KU252200	–	KU251833	KU251722	Wang et a. (2021)
*C. xanthorrhoeae*	ICMP 17903 ^T^	*Xanthorrhoea preissii*	Australia	JX010261	JX009927	JX009823	JX009478	JX010448	–	JX009653	KC790689	Weir et al. (2012)
*C. xishuangbannaense*	MFLUCC 19-0107 ^T^	*Magnolia liliifera*	China	MW346469	MW537586	MW660832	MW652294		–	–	–	Zhang et al. (2023)
*C. yuanjiangense*	YMF1.04996 ^T^	*Ageratina adenophora*	China	OK030885	OK513686	OK513583	OK513620	OK513649	–	–	–	Yu et al. (2022)
*C. yulongense*	CFCC 50818 ^T^	Vaccinium dunalianum var. urophyllum	China	MH751507	MK108986	MH793605	MH777394	MK108987	–	MH793604	PP861147	Zhang et al. (2023)
*C. boninense* (outgroup)	CBS 123755 ^T^	Crinum asiaticum var. sinicum	Japan	JQ005153	JQ005240	JQ005327	JQ005501	JQ005588	–	JQ005674	–	Damm et al. (2012)
*C. brasiliense* (outgroup)	CBS 128501 ^T^	*Passiflora edulis*	Brazil	JQ005235	JQ005322	JQ005409	JQ005583	JQ005669	–	JQ005756	–	Damm et al. (2012)
***Colletotrichum magnum* species complex**												
*C. brevisporum*	BCC 38876 ^T^	*Neoregelia* sp.	Thailand	JN050238	JN050227	–	JN050216	JN050244	–	–	–	Noireung et al. (2012)
*C. cacao*	CBS 119297 ^T^	*Theobroma cacao*	Costa Rica	MG600772	MG600832	MG600878	MG600976	MG601039	MG600916	–	–	Damm et al. (2019)
*C. guangdongense*	ZHKUCC 21-0105 ^T^	*Citrus maxima*	China	OL708415	OL855854	OL855864	OL855875	OL855885	ON315370	–	–	Liu et al. (2023)
*C. guangdongense*	ZHKUCC 21-0106	*Citrus maxima*	China	OL708420	OL855855	OL855865	OL855876	OL855886	–	–	–	Liu et al. (2023)
*C. kaifengense*	CAASZK33 ^T^	*Citrullus lanatus*	China	MZ475245	OL456715	OL901183	OL449313	OL456674	OM057724	–	–	Guo et al. (2022)
*C. kaifengense*	CAASZK32	*Citrullus lanatus*	China	MZ475244	OL456714	OL901182	OL449312	OL456673	OM057723	–	–	Guo et al. (2022)
*C. lobatum*	IMI 79736 ^T^	*Piper catalpaefolium*	Trinidad and Tobago	MG600768	MG600828	MG600874	MG600972	MG601035	MG600912	–	–	Damm et al. (2019)
*C. magnum*	CBS 519.97 ^T^	*Citrullus lanatus*	USA	MG600769	MG600829	MG600875	MG600973	MG601036	MG600913	–	–	Damm et al. (2019)
*C. magnum*	IMI391662	*Citrullus lanatus*	USA	MG600771	MG600831	MG600877	MG600975	MG601038	MG600915	–	–	Damm et al. (2019)
*C. magnum*	CBS575.97	*Citrullus lanatus*	USA	MG600770	MG600830	MG600876	MG600974	MG601037	MG600914	–	–	Damm et al. (2019)
*C. magnum*	CAASZK9	*Citrullus lanatus*	China	MZ475213	OL899046	OL901142	OL986316	OL988321	OM057685	–	–	Gou et al. (2022)
*C. magnum*	CAASZK34	*Citrullus lanatus*	China	MZ475246	OL899044	OL986134	OL986314	OL988319	OL986209	–	–	Gou et al. (2022)
*C. magnum*	CAASZKF13	*Citrullus lanatus*	China	MZ475166	OL456677	OL901145	OL449275	OL456636	OM057688	–	–	Gou et al. (2022)
*C. magnum*	CAASZK2	*Citrullus lanatus*, fruit	China	MZ475206	OL456679	OL901147	OL449277	OL456638	OM057690	–	–	Gou et al. (2022)
*C. magnum* (=*C. liaoningense*)	CGMCC3.17616, CAUOS2	Chili pepper	China	KP890104	KP890135	KP890127	KP890097	KP890111	–	–	–	Diao et al. (2016)
*C. magnum* (=*C. liaoningense*)	CAUOS6	Capsicum annuum var. conoides	China	–	–	KP890131	–	KP890115	–	–	–	Diao et al. (2017)
*C. magnum* (=*C. liaoningense*)	CAUOS3	Capsicum annuum var. conoides	China	KP890105	KP890136	KP890128	–	KP890112	–	–	–	Diao et al. (2017)
*C. magnum* (=*C. liaoningense*)	CAUOS4	Capsicum annuum var. conoides	China	KP890106	KP890137	KP890129	–	KP890113	–	–	–	Diao et al. (2017)
***C. magnum***	**GUCC 25-0029**	***Juglans regia***	**China**	**PV791818**	**PV979839**	**PV979806**	**PV979837**	**PV979907**	**PV979869**	–	–	**In this study**
***C. magnum***	**GUCC 25-0030**	***Juglans regia***	**China**	**PV791819**	**PV979840**	**PV979807**	**PV979838**	**PV979908**	**PV979870**	–	–	**In this study**
*C. merremiae*	CBS 124955 ^T^	*Merremia umbellata*	Panama	MG600765	MG600825	MG600872	MG600969	MG601032	MG600910	–	–	Damm et al. (2019)
*C. okinawense*	MAFF 240517 ^T^	Carica papaya, petiole	Japan	MG600767	MG600827	–	MG600971	MG601034	–	–	–	Damm et al. (2019)
*C. panamense*	CBS 125386 ^T^	*Merremia umbellata*	Panama	MG600766	MG600826	MG600873	MG600970	MG601033	MG600911	–	–	Damm et al. (2019)
*C. qilinense*	CAASZK13 ^T^	*Citrullus lanatus*, fruit	China	MZ475217	OL456694	OL901162	OL449292	OL456653	OM057703	–	–	Guo et al. (2022)
*C. qilinense*	CAASZK15	*Citrullus lanatus*	China	MZ475219	OL456696	OL901164	OL449294	OL456655	OM057705	–	–	Guo et al. (2022)
*C. dracaenophilum* (outgroup)	CBS 118199 ^T^	*Dracaena sanderana*	China	JX519222	JX546707	JX519230	JX519238	JX519247	JX546756	–	–	Damm et al. (2019)
*C. yunnanense* (outgroup)	CBS 13213 ^T^	*Buxus* sp.	China	JX546804	JX546706	JX519231	JX519239	JX519248	JX546755	–	–	Damm et al. (2019)

Ex-type/ex-epitype/ex-neotype/ex-lectotype strains are marked with ^T^; BRIP Queensland Plant Pathology Herbarium; CBSCBS-KNAW Fungal Biodiversity Centre, Utrecht, The Netherlands; CFCC China Forestry Culture Collection Center; CGMCC China General Microbiological Culture Collection Center; CMM Culture Collection of Phytopathogenic Fungi Prof. Maria Menezes, Federal Rural University of Pernambuco, Brazil; CNUCC Capital Normal University Culture Collection Center; CPCCulture collection of Pedro Crous, housed at CBS; GUCC the Plant Pathology Department of the College of Agriculture, Guizhou University, China; GZAAS Guizhou Academy of Agricultural Sciences, Guiyang, China; ICMP International Collection of Microorganisms from Plants; IMI International Mycological Institute; LC the LC Culture Collection (a personal culture collection of Lei Cai, housed in the Institute of Microbiology, Chinese Academy of Sciences); LGMF Culture Collection of Laboratory of Genetics of Microorganisms, Federal University of Parana, Curitiba, Brazil; MAFF the Genetic Resources Center, National Agriculture and Food Research Organization, Tsukuba, Ibaraki, Japan; MFLUMae Fah Luang University Herbarium Collection, Chiang Rai, Thailand; MFLUCCMae Fah Luang University Culture Collection, Chiang Rai, Thailand; NBRCNITE Biological Resource Center; NTUCCthe Department of Plant Pathology and Microbiology, National Taiwan University Culture Collection; RGM the Chilean Collection of Microbial Genetic Resources; SAUCCCulture collection of the Department of Plant Pathology, College of Plant Protection, Shenyang Agricultural University, China; VPRI the Victorian Plant Pathology Herbarium; YMF the Herbarium of the Laboratory for Conservation and Utilization of Bio-Resources, Yunnan University, Kunming, Yunnan, China; ZHKUCC the Culture Collection of Zhongkai University of Agriculture and Engineering. The strains generated in this study are in bold.

### ﻿Phylogenetic analyses

The raw readings were processed and organized into contigs using Geneious Prime 2025.0.3 Java Version 11.0.24+8 (64-bit) software (http://www.geneious.com). The newly generated sequences were used as queries to conduct a BLASTn search against the non-redundant (nr) database in GenBank. The retrieval of similar sequences was conducted, followed by the construction of numerous alignments. The GenBank taxonomy browser was utilized to verify all sequences classified as *Colletotrichum* in the database. BioEdit version 7.2.5 (Hall 1999) was used to assign open reading frames of the protein-coding sequences of *actin*, *gapdh*, *β-tubulin*, *chs-1*, and *cal* according to reference sequences in the GenBank database. The combined sequence data of all loci were used to perform maximum likelihood (ML), maximum parsimony (MP), and Bayesian posterior probability analysis (BI).

The dataset of each gene region was independently aligned with the ‘auto’ strategy (based on data size) in MAFFT (Katoh et al. 2019) and trimmed with the ‘gappyout’ method (based on gaps’ distribution) in TrimAl (Capella-Gutiérrez et al. 2009). BioEdit v. 7.0.9.0 (Hall 1999) was utilised for manual editing, where needed. The best-fit nucleotide substitution models for each dataset were selected based on the Bayesian information criterion (BIC) with rate heterogeneity by ModelFinder (Kalyaanamoorthy et al. 2017). Afterwards, all datasets were concatenated with partition information for the subsequent phylogenetic analyses.

Maximum likelihood (ML), maximum parsimony (MP) and Bayesian posterior probability (BI) analysis were performed using the CIPRES Science Gateway (https://www.phylo.org/portal2) (Miller et al. 2010). The maximum likelihood tree was constructed using RAxMLHPC2 on XSEDE with bootstrapping of 1000 replicates. The ML analysis utilized the GTR + GAMMA model. The maximum parsimony phylogenetic tree was performed using PAUP XSEDE (Swofford 2002). The Bayesian posterior probability (BI) analysis employed a Markov Chain Monte Carlo (MCMC) algorithm with MrBayes on XSEDE, involving four MCMC chains running for 1,000,000 generations and sampling at intervals of 100 generations. The first 25% of constructed trees were eliminated as burn-in, and the remaining trees were used to compute posterior probabilities (Ronquist et al. 2012). The resultant phylograms were visualised with FigTree v. 1.4.4 (Rambaut 2018) and formatted using Adobe Illustrator CC 22.0.0 (Adobe Systems, USA).

The Genealogical Concordance Phylogenetic Species Recognition (GCPSR) model with a pairwise homoplasy index (PHI) test was used to analyze the newly generated taxon and its most phylogenetically close neighbors (Quaedvlieg et al. 2014). The PHI test was performed in SplitsTree v. 4.14.6 (Huson 1998; Huson and Bryant 2006) with a five-locus concatenated dataset (ITS, *act*, *gapdh*, *β-tubulin*, and *chs-1*) to determine the recombination level among phylogenetically closely related species. A pairwise homoplasy index below a 0.05 threshold (Φw < 0.05) indicated the presence of significant recombination in the dataset. The relationship between closely related species was visualized by constructing a split graph.

## ﻿Results

Single-locus phylogenetic trees were initially generated for each gene region to assess individual tree topologies and clade support prior to constructing a combined gene tree. In this study, we introduce a novel *Colletotrichum* species and report 11 previously described species, with six representing new geographical and/or host records. Phylogenetic analyses were conducted using a comprehensive multilocus dataset, incorporating multi-gene regions depending on the species complex. Maximum Likelihood (ML), Maximum Parsimony (MP), and Bayesian posterior probability analysis (BI) methods were applied to infer phylogenetic relationships.

**Figure 1.**
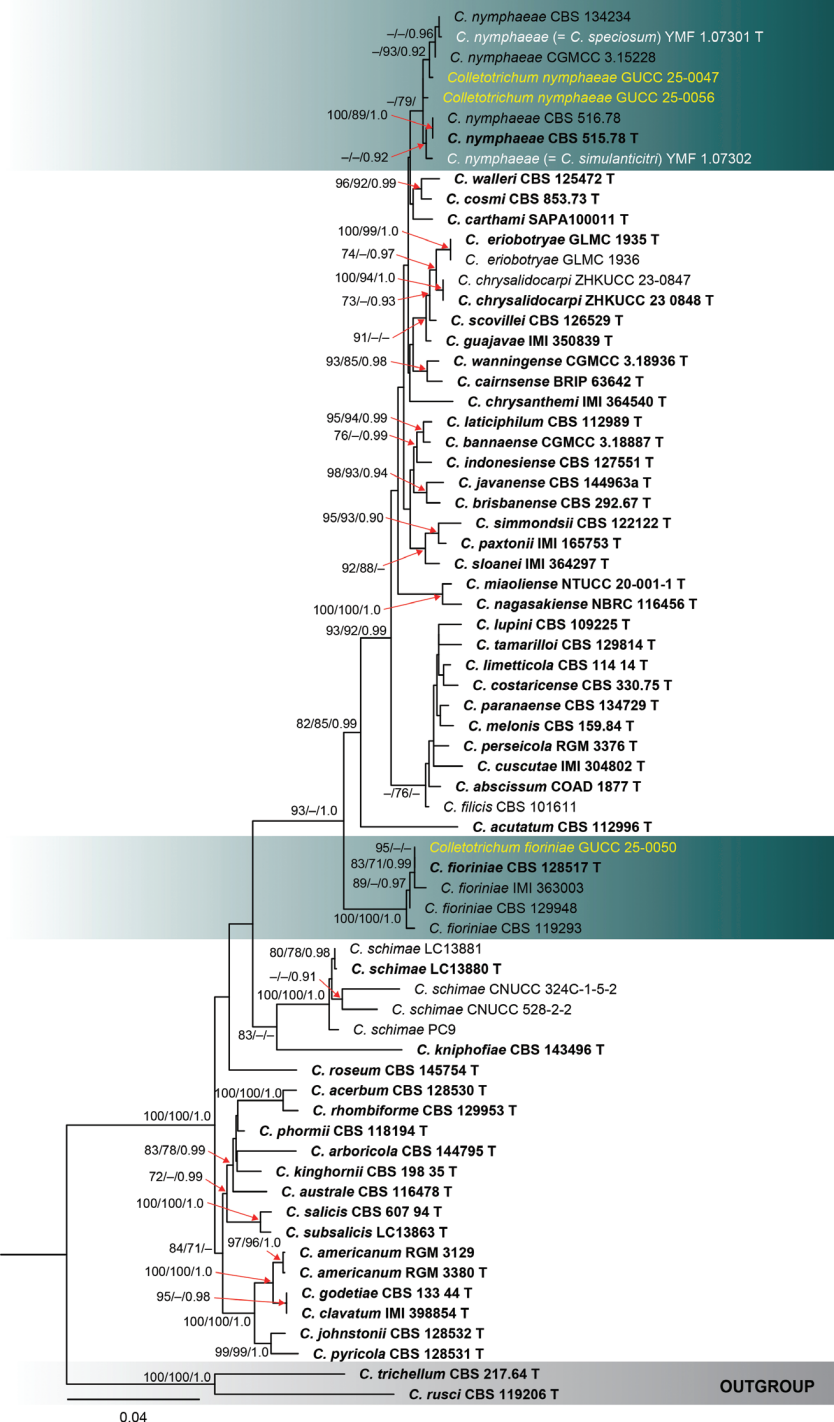
Phylogenetic tree constructed using a maximum likelihood (ML) analysis based on a combined ITS, *gapdh*, *chs-1*, *act*, *β-tubulin*, and *his3* sequences, representing *Colletotrichum acutatum* species complex. The tree topology of the ML analysis was identical to the Maximum Parsimony (MP) and Bayesian posterior probability (BI) analyses. The final RAxML tree with a likelihood value of -11021.830194 is presented here. The evolutionary model GTR+GAMMA was applied to all the genes. The analysis included sixty-nine (69) taxa with a total of 2183 characters, with 803 distinct alignment patterns, and 7.96% were gaps and undetermined characters. Estimated base frequencies were as follows: A = 0.224158, C = 0.310418, G = 0.240273, T = 0.225151; substitution rates AC = 1.652392, AG = 4.067151, AT = 1.193894, CG = 0.729619, CT = 7.723186, GT = 1.0; gamma distribution shape parameter *α* = 0.285275; tree length = 0.845774. Bootstrap support values for ML and MP ≥ 70% and Bayesian Posterior Probabilities (BI) ≥ 0.90 are indicated at the nodes as ML/MP/BI. The tree is rooted with *C. rusci* (CBS 119206) and *C. trichellum* (CBS 217.64). Type strains are denoted in bold and T and sequences generated in this study are in yellow. Bar = 0.04 represents the estimated number of nucleotide substitutions of site per branch.

The topologies resulting from the ML, MP, and BI analyses were congruent, with no significant conflicts observed among the trees generated by the three methods. The combined analyses resolved the newly obtained strains within five *Colletotrichum* species complexes: *C. acutatum*, *C. bambusicola*, *C. trichellum*, *C. gloeosporioides*, and *C. magnum*. In each case, the isolates clustered with previously accepted members of the respective complexes, and the resulting trees exhibited strong nodal support, as shown in Figs 1, 3, 5, 12.

One novel species, *Colletotrichum macroconidii* (strain GUCC 25-0028 and GUCC 25-0063), was recovered as a distinct lineage within the *gloeosporioides* species complex. This taxon is supported by bootstrap values and Bayesian posterior probabilities, confirming its phylogenetic distinctiveness. In addition, 11 known species were identified from our collections. Among them, six species represent new records for China or for specific host associations: *C. fioriniae* from *Parthenocissus tricuspidata*, *C. trichellum* from *Hedera* spp., *C. juglandicola* from *Camellia japonica* and *Ilex* spp., *C. nanhuaense* from *Juglans regia*, *C. jiangxiense* from *Juglans regia*, and *C. magnum* from *Juglans regia*. The remaining five species: *C. nymphaeae*, *C. metake*, *C. fructicola*, *C. siamense*, and *C. gloeosporioides* are recognized taxa that were previously reported in similar or nearby regions. Detailed phylogenetic placements and morphological characteristics of all taxa are provided in the respective species descriptions and notes.

**Figure 2.**
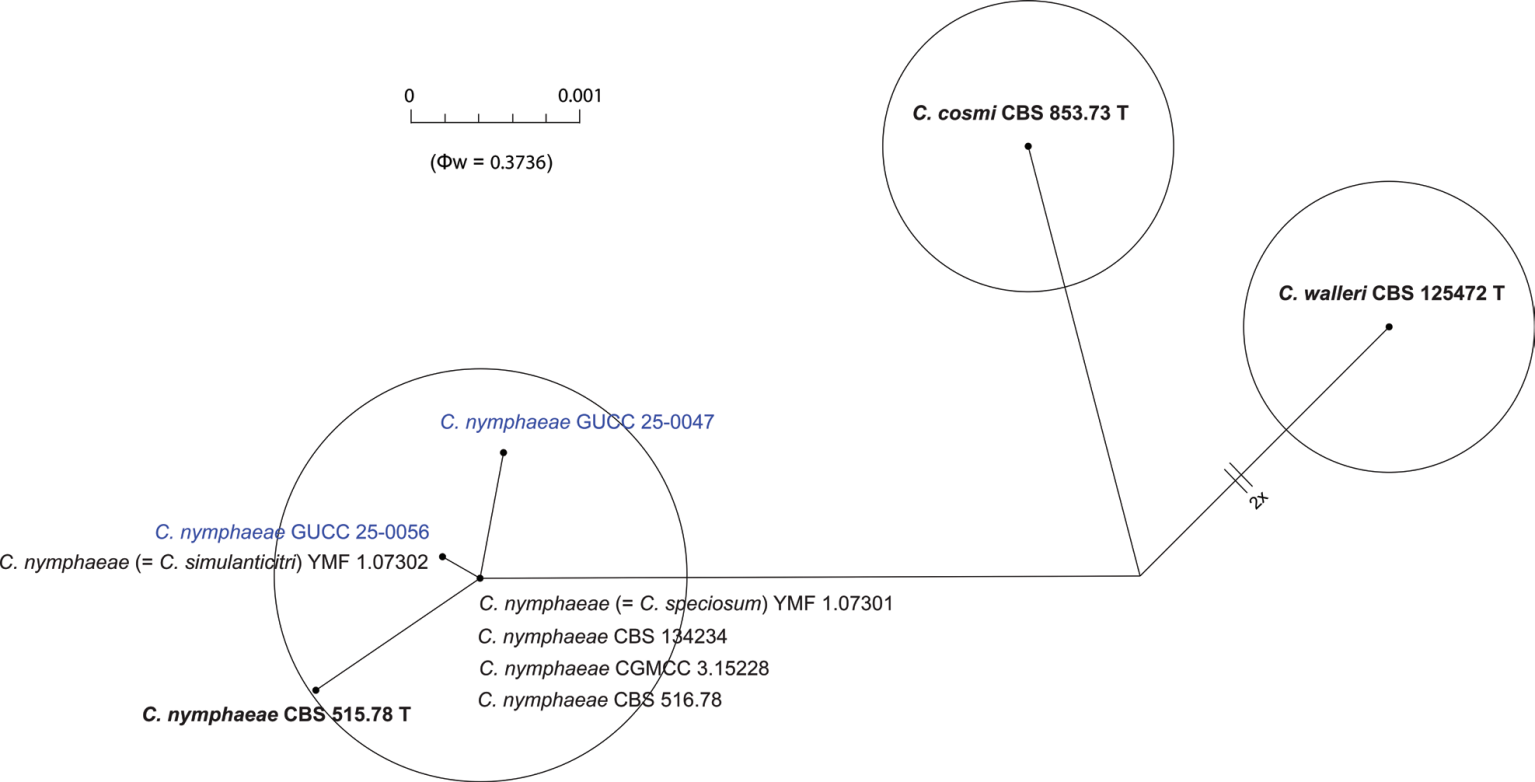
The results of the pairwise homoplasy index (PHI) test for closely related species of *Colletotrichum nymphaeae* in this study using both LogDet transformation and splits decomposition. PHI test results (Φw) > 0.05 indicate no significant recombination within the dataset.

To further validate the taxonomic independence of the novel taxon, the Genealogical Concordance Phylogenetic Species Recognition (GCPSR) criterion was applied. The Pairwise Homoplasy Index (PHI) test was employed to detect recombination events among closely related taxa. A Φw value below 0.05 indicates significant recombination, while values above this threshold support genetic separation (Figs 2, 6). For the new species *C. macroconidii*, the PHI test produced non-significant values (Φw = 1.0), indicating no detectable recombination with the closely related species, *C. gardeniae* (Fig. 6). These results support the recognition of *C. macroconidii* as a phylogenetically and evolutionarily distinct species.

**Figure 3.**
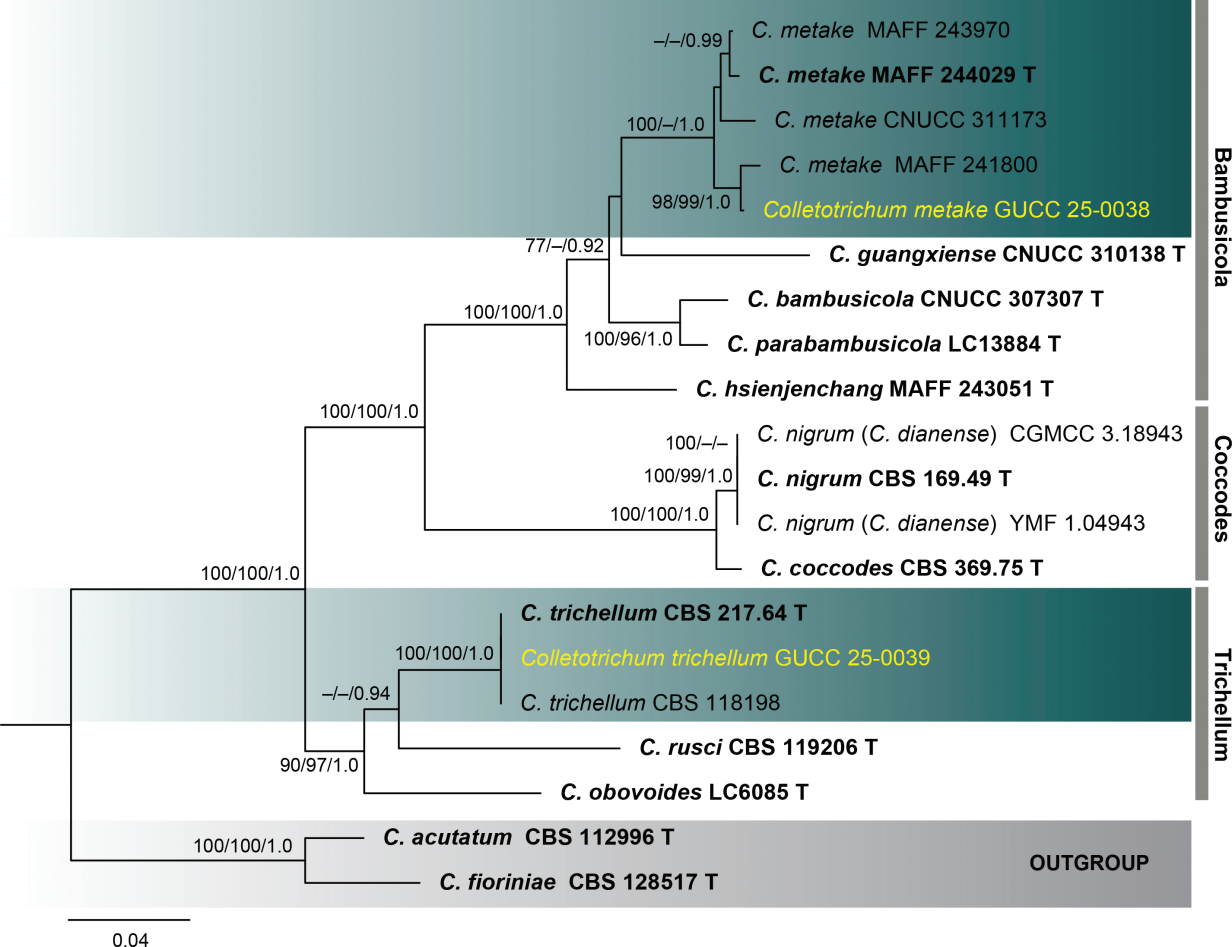
Phylogenetic tree constructed using a maximum likelihood (ML) analysis based on a combined ITS, *gapdh*, *chs-1*, *act*, *β-tubulin*, and *his3* sequences, representing *Colletotrichum bambusicola*, *coccodes*, and *trichellum* species complexes. The tree topology of the ML analysis was identical to the Maximum Parsimony (MP) and Bayesian posterior probability (BI) analyses. The final RAxML tree with a likelihood value of -9417.584651 is presented here. The evolutionary model GTR+GAMMA was applied to all the genes. The analysis included twenty (20) taxa with a total of 2341 characters, with 764 distinct alignment patterns, and 21.79% were gaps and undetermined characters. Estimated base frequencies were as follows: A = 0.217665, C = 0.311394, G = 0.244784, T = 0.226158; substitution rates AC = 1.493066, AG = 4.079410, AT = 1.470090, CG = 1.247573, CT = 7.473294, GT = 1.0; gamma distribution shape parameter *α* = 0.267361; tree length = 0.838159. Bootstrap support values for ML and MP ≥ 70% and Bayesian Posterior Probabilities (BI) ≥ 0.90 are indicated at the nodes as ML/MP/PP. The tree is rooted with *C. acutatum* (CBS 112996) and *C. fioriniae* (CBS 128517). Type strains are denoted in bold and T and sequences generated in this study are in yellow. Bar = 0.04 represents the estimated number of nucleotide substitutions of site per branch.

### ﻿ Colletotrichum fioriniae

Taxon classificationFungiGlomerellalesGlomerellaceae

(Marcelino & Gouli) Pennycook, Mycotaxon 132(1): 150 (2017) [2016]

7ED6EA67-6CC3-5AF3-A21D-300A598AF318

#### Description.

See Damm et al. (2012a).

#### Material examined.

China • Guizhou Province, Tongren, *Parthenocissus tricuspidata* (Siebold & Zucc.) Planch., 2024.07.11, coll. Gou Shiqi, D94/GSQ/GZ38 (dried culture, HGUP 25-0036), living culture GUCC 25-0050.

#### Notes.

*Parthenocissus tricuspidata* (Vitaceae, Vitales) is a vigorous liana widely cultivated for ornamental and ecological purposes due to its exceptional adaptability to various environmental conditions, including low temperatures, drought, and nutrient-poor soils (Steinbrecher et al. 2011). Its strong adhesive and climbing abilities make it a preferred species for covering walls, pavilions, and rocks (He et al. 2011). However, with its increasing use in landscape greening, the plant has become more susceptible to a growing number of pathogenic threats. Recent studies have documented several fungal pathogens associated with *P. tricuspidata*, including *Phyllosticta partricusidatae* (Zhou et al. 2015), *Colletotrichum siamense* (Zhao et al. 2020), *Septoria tormentillae* (Wang et al. 2020), *Diaporthe tulliensis* (Huang et al. 2021a), *Coniella vitis* (Yin et al. 2023), *Neophysopella vitis* (Zhou et al. 2024), *C. gloeosporioides*, and *C. siamensethe* (Wang et al. 2025), highlighting the plant vulnerability to fungal diseases.

In the present study, the fungal strain GUCC 25-0050 was isolated from symptomatic leaves of *P. tricuspidata*. Based on multilocus phylogenetic analyses of ITS, *gapdh*, *chs-1*, *act*, *β-tubulin*, and *his3* gene regions (Fig. 1), this strain was identified as *Colletotrichum fioriniae*. This identification is supported by its phylogenetic placement. This is the first report of *C. fioriniae* on *P. tricuspidata* in China. The identification of *C. fioriniae* as a pathogen of *P. tricuspidata* underscores the need for further investigation into its epidemiology, pathogenicity, and potential impact on ornamental and ecological plantings.

### ﻿ Colletotrichum nymphaeae

Taxon classificationFungiGlomerellalesGlomerellaceae

(Pass.) Aa, Netherlands Journal of Plant Pathology, Supplement 1 84(3): 110 (1978)

AFAAD7F4-9DE0-5748-87FF-65CD75D06835

= Colletotrichum speciosum Z.F. Yu, in Yu et al, J. Fungi 8(2, no. 185): 24 (2022).
= Colletotrichum simulanticitri Z.F. Yu, in Yu et al, J. Fungi 8(2, no. 185): 24 (2022).

#### Description.

See more details in Damm et al. (2012a).

#### Material examined.

China • Guizhou Province, Tongren, on unidentified branch, 2024.07.11, coll. Gou Shiqi, D79/FDS28/GZ35 (dried culture, HGUP 25-0033), living culture GUCC 25-0047; *ibid*., on rotten branch, 2024.07.11, coll. Gou Shiqi, E43/FDS8/GZ45 (dried culture, HGUP 25-0040), living culture GUCC 25-0056.

#### Notes.

This species was originally described from *Nymphaea alba* leaves in Kortenhoef by Van der Aa (1978). According to Damm et al. (2012a), *C. nymphaeae* is reliably distinguished from related taxa by the beta tubulin (*β-tubulin*) gene, although other loci show high intraspecific variability. Based on morphological characteristics and multilocus phylogenetic analyses, strains GUCC 25-0047 and GUCC 25-0056 were identified as *Colletotrichum nymphaeae*. Morphologically, the new strain in this study produces an asexual morph on PDA with hyaline to pale brown, septate, branched, and smooth-walled conidiophores. The conidiogenous cells were cylindrical, measuring 9.7–15 × 2.8–4.3 µm, sharing similar coloration and wall texture, and were characterized by a distinct collarette and evident periclinal thickening. Conidia were hyaline, aseptate, smooth-walled, and straight, varying from cylindrical to cylindric-clavate, measuring 9.9–15.5 × 3.5–5 µm, consistent with the characteristics of the genus *Colletotrichum*. Phylogenetically, both strains clustered with *C. nymphaeae*, showing high sequence similarity and revealed low genetic differences for each gene with the type strain (CBS 515.78): GUCC 25-0047 (ITS = 99% [2/541], *gapdh* = 98% [5/248], *chs-1* = 99% [1/282], *act* = 99% [1/246], *β-tubulin* = 99% [1/490], *his3* = 99% [2/382]) and GUCC 25-0056 (ITS =99% [3/541], *gapdh* = 99% [3/248], *chs-1* = 99% [1/282], *act* = 100% [0/246], *β-tubulin* = 100% [0/490], *his3* = 99% [2/382]). These results indicate only minor genetic variation from *C. nymphaeae*. The species formed a well-supported clade, clearly distinct from other taxa within the *C. acutatum* species complex (Fig. 1). This placement was further supported by the PHI test, which indicated significant recombination among the two collections (Fig. 2).

Additionally, *Colletotrichum speciosum* (YMF 1.07301) and *Colletotrichum simulanticitri* (YMF 1.07302) clustered with the type strain of *C. nymphaeae* (CBS 515.78), along with five other strains, including GUCC 25-0047 and GUCC 25-0056 (Fig. 1). Phylogenetic analyses based on single loci showed that *chs-1*, *β-tubulin*, and *his3* provided the highest resolution for this species complex, producing a topology most consistent with the combined multilocus tree and supported by significant bootstrap values. *Colletotrichum speciosum* was described by Yu et al. (2022) based only on ITS sequence data, which shows 99% similarity (538/541 bp) with *C. nymphaeae*, suggesting a close relationship. However, since only ITS is not sufficient for species delimitation in *Colletotrichum*, and only a single strain is known, we tentatively consider *C. speciosum* a synonym of *C. nymphaeae*. Further studies using multilocus genes and more strains are needed to confirm this synonymy. In comparison, *C. simulanticitri* was described using four loci (ITS, *gapdh*, *chs-1*, and *act*), providing stronger molecular support (Yu et al. 2022). Both the combined analysis and single-locus trees placed *C. simulanticitri* together with *C. nymphaeae* strains. Pairwise comparisons between *C. simulanticitri* (YMF 1.07302) and the type strain of *C. nymphaeae* (CBS 515.78) revealed low genetic differences: ITS = 0.3% (2/542 bp), *gapdh* = 0.8% (2/238 bp), *chs-1* = 0% (0/226 bp), and *act* = 0% (0/246 bp). Morphologically, *C. simulanticitri* shares similarities with *C. nymphaeae*, including hyaline, cylindrical to oblong conidia (10–13.5 × 4–5 µm for *C. simulanticitri*), as well as dark brown, septate, clavate, or oval appressoria (Damm et al. 2012a; Yu et al. 2022). Notably, the morphological characters of *C. simulanticitri* were observed on CMA, whereas *C. nymphaeae* was observed on PDA in this study. Taken together, the close phylogenetic position, minimal genetic divergence, and morphological similarity strongly indicate that *C. simulanticitri* and *C. nymphaeae* represent the same species. This is further confirmed by the PHI test, which indicated significant recombination between the taxa (Fig. 2). It is also important to note that both *C. speciosum* and *C. simulanticitri* are currently listed as nomen invalidum (nom. inval.) in MycoBank. The name *C. simulanticitri* has already been synonymized with *C. nymphaeae*, and *C. speciosum* is invalid due to a violation of Article 40.8 of the International Code of Nomenclature. Based on morphological features, multilocus phylogeny, recombination analysis, and taxonomic status, we recognize *C. simulanticitri* as a synonym of *C. nymphaeae*, and provisionally treat *C. speciosum* as its synonym as well.

### ﻿ Colletotrichum metake

Taxon classificationFungiGlomerellalesGlomerellaceae

Sacc., Annls mycol. 6(6): 557 (1908)

34378B8C-48A3-5C43-9203-00EA7751A640

#### Description.

See Sato et al. (2012).

#### Material examined.

CHINA • Guizhou Province, Guiyang, symptomatic leaves of Poaceae spp. (bamboo), 2024.01.13, coll. Wang Xingchang, BB-1-19-1-4-A/GZ26 (dried culture, HGUP 25-0026), living culture GUCC 25-0038.

#### Notes.

In China, research on *Colletotrichum* species infecting bamboo remains scarce. For instance, Ren et al. (2008) identified *C. coccodes* as the causal pathogen of anthracnose on *Bambusa pervariabilis*. More recently, Wang et al. (2021a) reported *C. metake* as a seed endophyte from *Chimonobambusa quadrangularis*. Despite the high diversity of bamboo hosts, *Colletotrichum* species associated with bamboo are relatively understudied. To date, only five *Colletotrichum* species have been reported from members of the Bambusoideae subfamily. Among these, *C. coccodes* (Ren et al. 2008), *C. graminicola* (Parris 1959), and *C. trichellum* (Crouch et al. 2009) were identified based on morphological characteristics. In contrast, *C. hsienjenchang* and *C. metake* were described using a combination of morphological and phylogenetic analyses (Sato et al. 2012; Wang et al. 2021a). Additionally, Zhou (2018) investigated the endophytic fungal communities in bamboo seeds and found *Colletotrichum* to be one of the dominant genera, although the isolates were not identified to species level.

In the present study, strain GUCC 25-0038, isolated from bamboo in Guizhou Province, was identified as *Colletotrichum metake* based on multilocus phylogenetic analyses (Fig. 3). This represents a novel strain of *C. metake*, which was previously reported from bamboo in China by Wang et al. (2021a).

### ﻿ Colletotrichum trichellum

Taxon classificationFungiGlomerellalesGlomerellaceae

(Fr.) Pat., Cat. Rais. Pl. Cellul. Tunisie (Paris): 127 (1897)

AFD619EA-5280-5BFD-BD81-022E6AE3EC64

Fig. 4

#### Description.

See Sutton (1962).

#### Material examined.

CHINA • Guizhou Province, Guiyang, Guizhou University, symptomatic leaves of *Hedera* L. (Ivy), 2024.01.20, coll. Wang Xingchang, 1.28cct1-2/GZ27 (dried culture, HGUP 25-0027), living culture GUCC 25-0039.

#### Notes.

*Colletotrichum trichellum* is known to cause leaf and stem spots on English ivy (*Hedera helix*) and has been reported as a pathogen in several countries, including Canada, Germany, Guatemala, the Netherlands, New Zealand, and the United Kingdom (Damm et al. 2009; Jayawardena et al. 2021). This species is particularly notable for its ability to infect *Hedera* species, producing distinct symptoms such as necrotic lesions and chlorosis on leaves and stems. In the present study, strain GUCC 25-0039, isolated from *Hedera* spp. (ivy), was identified as *Colletotrichum trichellum* based on both morphological characteristics and multilocus phylogenetic analyses. Morphologically, the strain in this study produced an asexual morph on oatmeal agar (OA) with acervular conidiomata that appeared dark and were surrounded by mycelium, bearing white to cream conidial masses; conidiophores were hyaline, smooth-walled, septate, and branched, while setae were medium to dark brown, smooth to finely verruculose, with a knobbed and rounded tip, 1–3-septate, and 25–50 µm long. Conidiogenous cells were subcylindrical, straight to curved, and measured 16.4–26.4 × 2.6–4.4 µm. Conidia were hyaline, aseptate, falcate, slightly curved, fusiform, and abruptly tapered at both ends, measuring 20.4–25.7 × 3.8–5.5 µm. The overall morphology is consistent with that of *C. trichellum* as previously reported (Sutton 1962; Damm et al. 2009). Phylogenetic analyses of combined gene regions placed strain GUCC 25-0039 within the well-supported *C. trichellum* clade (Fig. 3). The multilocus phylogenetic tree showed a clear monophyletic grouping of GUCC 25-0039 with reference strains of *C. trichellum*, supported by 100% ML, 100% MP, 1.00 BI, confirming its taxonomic identity within the *Colletotrichum trichellum* species complex. Morphologically, the strain exhibited characteristics consistent with those described for *C. trichellum*, including simple to branched conidiophores and hyaline, fusiform, aseptate conidia with slight curvature, which are features considered diagnostic for this species (Sutton 1962; Damm et al. 2009). Additionally, the presence of elongated, acicular conidia further supported its placement within the genus *Colletotrichum*. Given the pathogenic potential of *C. trichellum* on ornamental *Hedera* species and its widespread distribution, its detection poses a potential concern for the horticultural industry. This study represents the first geographical record of *C. trichellum* associated with *Hedera* spp. in China.

**Figure 4.**
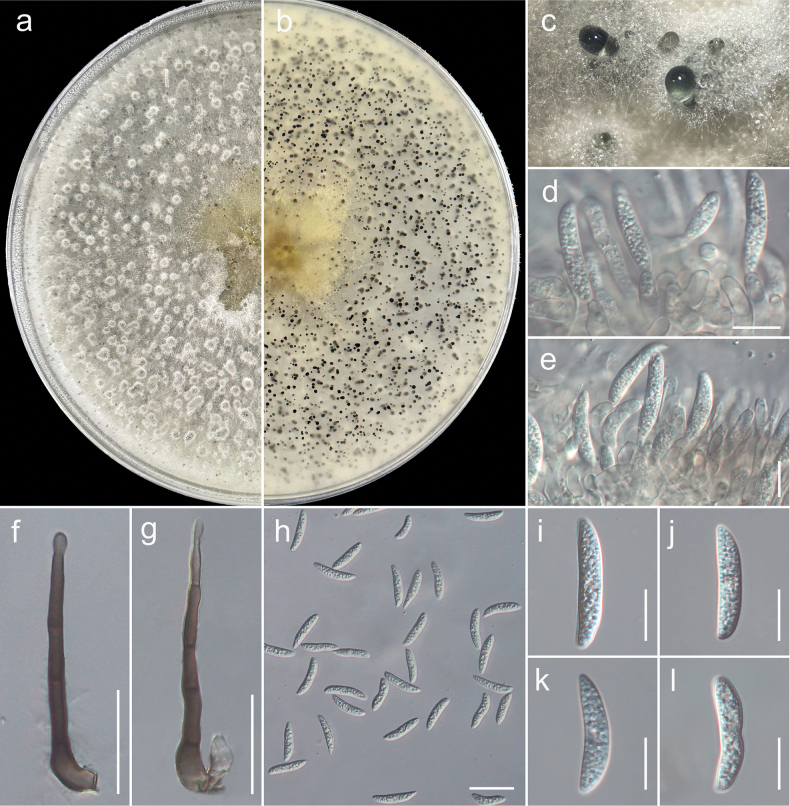
*Colletotrichum trichellum* (GUCC 25-0039, new record). a, b. Culture on OA (a-above, b-reverse); c. Conidiomata; d, e. Conidiogenous cells giving rise to conidia; f, g. Setae; h–l. Conidia. Scale bars: 10 µm (d, e, i–l); 25 µm (f–h).

**Figure 5.**
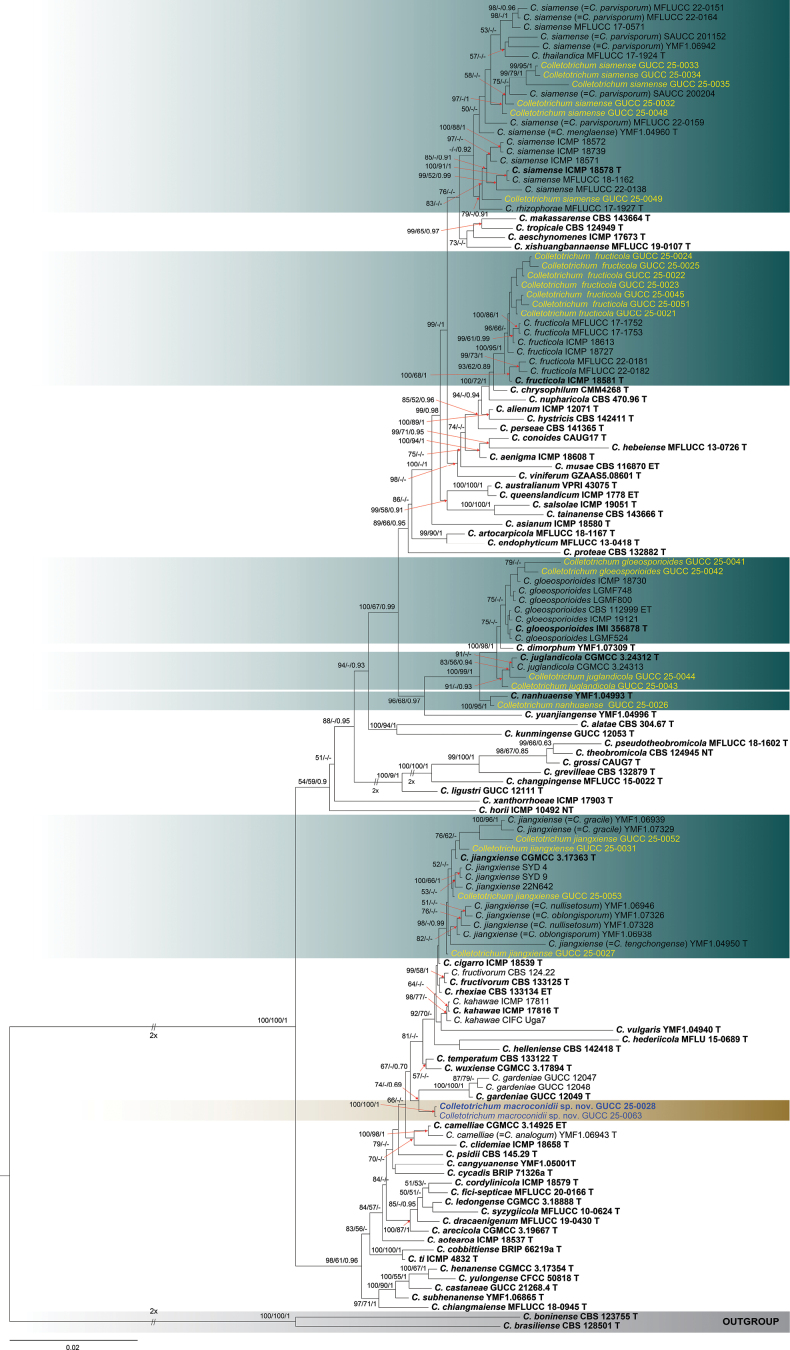
Phylogenetic tree constructed using a Bayesian posterior probability (BI) analysis based on a combined ITS, *gapdh*, *act*, *chs-1*, *β-tubulin*, and *cal* sequences, representing *Colletotrichum gloeosporioides* species complex. The tree topology of the ML analysis was identical to the Maximum Parsimony (MP) and Bayesian posterior probability (BI) analyses. The final RAxML tree with a likelihood value of -11975.336 is presented here. The evolutionary model GTR+GAMMA was applied to all the genes. The analysis included one hundred ninety-five (195) taxa with a total of 1689 characters, with 852 distinct alignment patterns, 495 parsimony-informative, 192 singleton sites, and 1002 constant sites. Estimated base frequencies were as follows: A = 0.229358, C = 0.298987, G = 0.241845, T = 0.226158; substitution rates AC = 1.085133, AG = 3.378568, AT = 1.335103, CG = 0.972042, CT = 5.776668, GT = 1.0; gamma distribution shape parameter *α* = 0.371036; tree length = 1.239. Bootstrap support values for ML and MP ≥ 50% and Bayesian Posterior Probabilities (BI) ≥ 0.90 are indicated at the nodes as ML/MP/BI. The tree is rooted with *C. acidae* (MFLUCC 17-2659) and *C. truncatum* (CBS 151.35). Type strains are denoted in bold and T and sequences generated in this study are in yellow. Bar = 0.03 represents the estimated number of nucleotide substitutions of site per branch.

**Figure 6.**
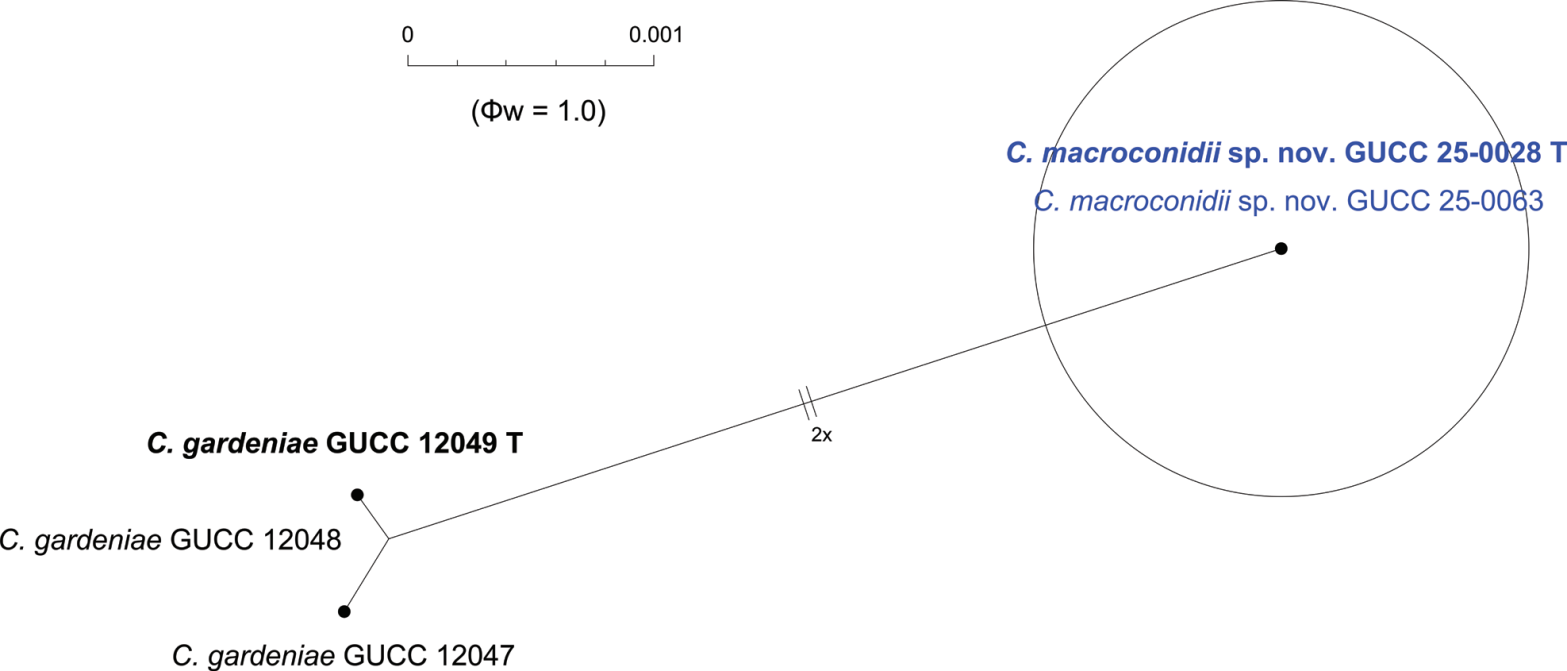
The results of the pairwise homoplasy index (PHI) test for closely related species of *Colletotrichum macroconidii* sp. nov. in this study using both LogDet transformation and splits decomposition. PHI test results (Φw) > 0.05 indicate no significant recombination within the dataset.

### ﻿ Colletotrichum macroconidii

Taxon classificationFungiGlomerellalesGlomerellaceae

Norph. & M.T. Zou sp. nov.

99741D26-19A0-5F80-9596-59580E10F947

903940

Figs 7, 8

#### Etymology.

The epithet ‘macroconidii’ refers to the distinctly large conidia produced by this species, which serve as a key morphological feature distinguishing it from closely related taxa in the *Colletotrichum gloeosporioides* species complex. Derived from Greek *makros* (large) and Latin *conidium* (spore), meaning having large conidia.

#### Holotype.

HGUP 25-0018.

#### Ex-type.

GUCC 25-0028.

#### Description.

***Associated*** with symptomatic leaves of *Juglans regia* L. ***Sexual morph***: Not observed. ***Asexual morph*: *Conidiomata*** pycnidial, globose, dark brown, superficial on WA, releasing conidia in a yellow mass, slimy from which setae and conidiophores were produced. ***Setae*** dark brown, concoloured, smooth-walled to finely verruculose, 1–3-septate, (80–)100–150(–180) µm long, base cylindrical to conical, 4–6(–9) µm diam., tip acute to roundish. ***Chlamydospores*** in cultures observed, in branched chains, brown, verrucose, 11–25 × 5–8 µm. ***Conidiophores*** pale brown, septate, strongly branched, smooth-walled or verrusculose, up to 90 µm long. ***Conidiogenous cells*** enteroblastic, pale brown, smooth-walled or verruculose, cylindrical to elongate ampulliform, 10–25 × 3–5 µm, opening 1.5–2 µm diam, collarette distinct, 1–2 µm long, periclinal thickening visible. ***Conidia*** (14.4–)15–17(–18.2) × (4.5–)5–6(–6.8) μm (mean ± SD = 16 ± 0.4 × 5 ± 0.9 μm), n = 80, L/W ratio = 2.8, hyaline, aseptate, straight, smooth-walled, cylindrical, the apex and base rounded, guttules contents with 1–2 large drop.

#### Culture characteristics.

Colonies on PDA reaching 7–8 cm diam after 7 d at room temperature (±25 °C), under light 12 hr/dark 12 hr, colonies rhizoid to filamentous, dense, flat or raised surface, with filiform margin, white from above and white to pale-yellow reverse, with producing grouped-pycnidia. Colonies on WA with/without sterilized toothpick, reaching 5 cm in diameter after 7 d at room temperature (±25 °C), under light 12 hr/dark 12 hr, colonies rhizoid to filamentous, dense, with flat surfaces and filiform margins, appearing white to pale gray-green from both the surface and reverse. Pycnidia developed both on the agar surface and as immersed structures within the medium.

#### Material examined.

China • Yunnan Province, Dali, Symptomatic leaves of *Juglans regia* L., 2023.11.12, coll. Zou Mengting, DJ5-7/GZ12_1, (dried culture, HGUP 25-0018, ***holotype***), ex-type living culture GUCC 25-0028; *ibid*., GZ12_2, living culture GUCC 25-0063.

#### Notes.

**Figure 7.**
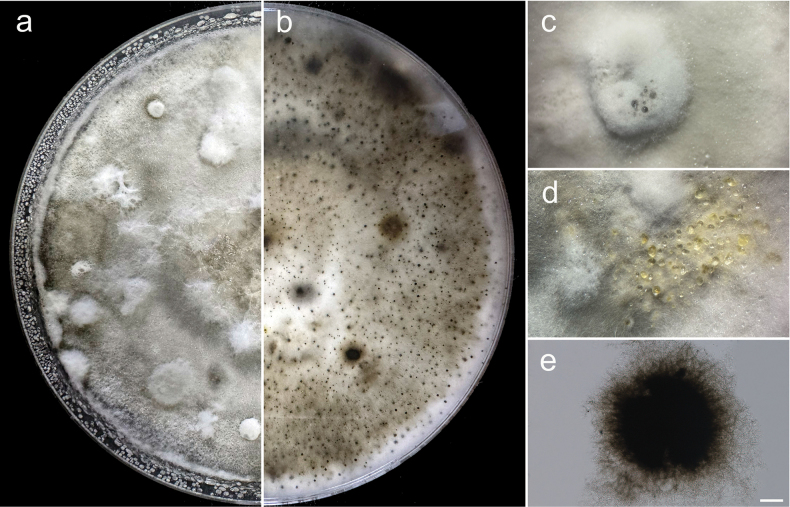
*Colletotrichum macroconidii* (GUCC 25-0028, ex-type). a, b. Culture on PDA 10-days (a-above, b-reverse); c, d. Conidiomata on PDA; e. Conidioma immersed in PDA. Scale bars: 10 µm (e).

Within the *Colletotrichum gloeosporioides* species complex (Fig. 5), two strains (GUCC 25-0028 and GUCC 25-0063) formed a distinct sister clade to *C. gardeniae* with 74% ML, 0.69 BI support (Fig. 5). Morphologically, *C. macroconidii* exhibits typical features of the complex, including hyaline, smooth-walled, aseptate, straight, cylindrical conidia with rounded apices and bases. However, it can be distinguished from its closest relatives by subtle yet consistent differences in conidial morphology. In particular, this species produces narrower conidia compared to *C. gardeniae*, associated with leaf spots on *Gardenia jasminoides*, which has conidia measuring 16.2 ± 0.8 × 6.1 ± 0.4, with a length/width (L/W) ratio of 2.7. In addition, *C. macroconidii* produces setae, which were not observed in *C. gardeniae* (Zhang et al. 2023b). It is important to note that the micromorphological characteristics of *C. gardeniae* were obtained from cultures grown on PDA, whereas those of *C. macroconidii* were observed on WA, as the latter species did not sporulate on PDA. On PDA, *C. gardeniae* formed flat colonies with an entire margin, white to vinaceous buff in color, turning orange in the center due to sporulation, and partially covered by short white aerial mycelium, with a growth rate of 72 mm in 7 days (Zhang et al. 2023b). In contrast, *C. macroconidii* produced rhizoid to filamentous, dense colonies with flat to raised surfaces and filiform margins. Colonies were white from above and white to pale-yellow in reverse, forming grouped pycnidia; however, no sporulation was observed within 7–10 days (Fig. 7). In addition to morphological differentiation, phylogenetic analysis based on multilocus sequence data supports the distinctiveness of *C. macroconidii*. Sequence similarity between these two species revealed differences of 3/547 base pairs in the ITS region, 4/231 base pairs in the *gapdh* region, 2/249 base pairs in the *chs-1* region, no differences in the act region, and 5/689 base pairs in the *β-tubulin* region. The *C. gardeniae* was missing *his3*, *Apmat*, and *cal* gene sequences. The pairwise homoplasy index (PHI) test showed no significant evidence of recombination between *C. macroconidii* and *C. gardeniae* (Φw = 1.0000; Fig. 6), further confirming its genetic separation. Taken together, the combination of morphological and the absence of recombination supports the recognition of *Colletotrichum macroconidii* as a novel species within the *C. gloeosporioides* species complex.

**Figure 8.**
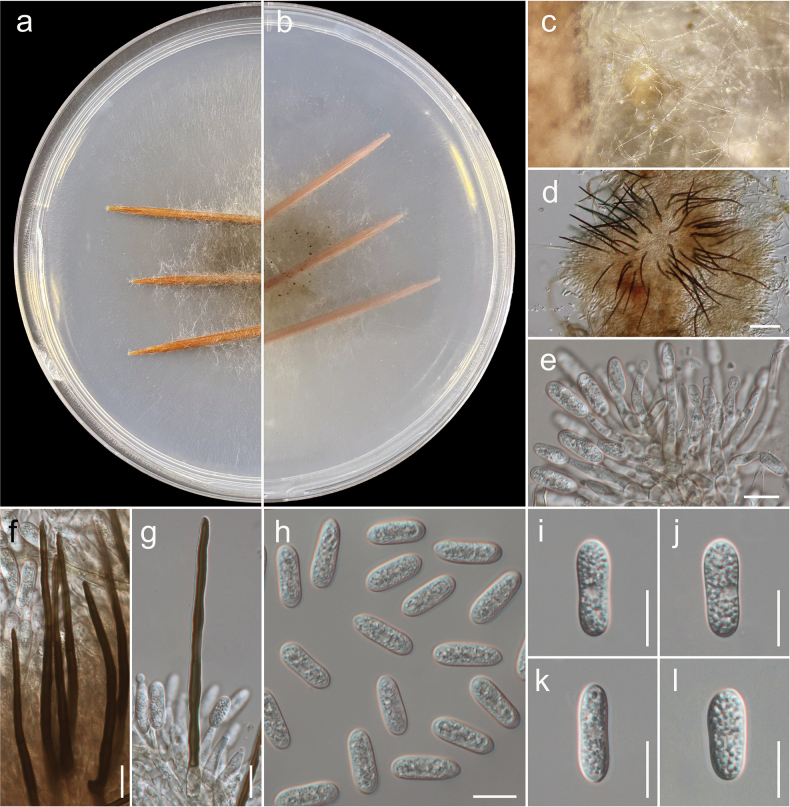
*Colletotrichum macroconidii* (GUCC 25-0028, ex-type). a, b. Culture on WA with sterile toothpick 10-days (a-above, b-reverse); c. Conidiomata on WA; d. Setose conidioma; e. Conidiophores, conidiogenous cells, giving rise to conidia; **f, g.** Setae; h–l. Conidia. Scale bars: 50 µm (d); 10 µm (e–l).

### ﻿ Colletotrichum fructicola

Taxon classificationFungiGlomerellalesGlomerellaceae

Prihast., L. Cai & K.D. Hyde, Fungal Diversity 39: 96 (2009)

B6C5FE05-0C61-5C79-868B-0B8F21CF0F9B

#### Description.

See Wang et al. (2017).

#### Material examined.

China • Yunnan Province, Dali, on symptomatic fruit of *Juglans regia* L., 2023.11.12, coll. Zou Mengting, DJ11-9/GZ05 (dried culture, HGUP 25-0011), living culture GUCC 25-0021; • *ibid*. symptomatic fruit of *Juglans regia* L., 2023.11.12, coll. Zou Mengting, DJ1-6/GZ06 (dried culture, HGUP 25-0012), living culture GUCC 25-0022; • *ibid*. symptomatic fruit of *Juglans regia* L., 2023.11.12, coll. Zou Mengting, DJ18-4/GZ07 (dried culture, HGUP 25-0013), living culture GUCC 25-0023; • *ibid*. symptomatic fruit of *Juglans regia* L., 2023.11.12, coll. Zou Mengting, DJ21-6/GZ08 (dried culture, HGUP 25-0014), living culture GUCC 25-0024; • *ibid*. symptomatic fruit of *Juglans regia* L., 2023.11.12, coll. Zou Mengting, DJ5-5/GZ09 (dried culture, HGUP 25-0015), living culture GUCC 25-0025; CHINA, Guizhou Province, Tongren, on symptomatic leaves of Actinidia chinensis var. deliciosa (Kiwi Fruit Leaf), 2024.07.11, coll. Gou Shiqi, D20/FDS1/GZ33 (dried culture, HGUP 25-0032), living culture GUCC 25-0045; • *ibid*. on symptomatic leaves of *Rosa chinensis* Jacq., 2024.07.11, coll. Gou Shiqi, D97/FDS23ROSE/GZ39 (dried culture, HGUP 25-0037), living culture GUCC 25-0051.

#### Notes.

*Colletotrichum fructicola* has been increasingly recognized as a significant plant pathogen with a broad host range in China. In 2017 (Wang et al. 2017), it was identified as the pathogen of walnut anthracnose on *Juglans regia* in Shandong Province, where infected fruits exhibited subcircular to irregular sunken lesions with pink conidial masses. The identification was confirmed through morphological characteristics and molecular analyses (Wang et al. 2017). *Colletotrichum fructicola* was also reported as the causal agent of leaf spot disease on *Actinidia chinensis*, based on morphological characteristics and multilocus phylogenetic analyses (Huang et al. 2022). Recently, *C. fructicola* was associated with anthracnose on *Rosa chinensis* in Henan Province, presenting as irregular brown specks that expanded into large necrotic lesions. Pathogenicity assays and molecular data supported its identification (Du et al. 2023). Peng et al. (2023) reported that among *Colletotrichum* species in China, *C. fructicola* is the dominant taxon causing *Camellia* anthracnose, making it the most prevalent species on *Camellia* spp. These findings emphasize the expanding distribution and host range of *C. fructicola* in China.

In the present study, strains GUCC 25-0021, GUCC 25-0022, GUCC 25-0023, GUCC 25-0024, and GUCC 25-0025, isolated from *Camellia japonica*, as well as GUCC 25-0045 and GUCC 25-0051, obtained from *Actinidia chinensis* and *Rosa chinensis*, respectively, were identified as *C. fructicola* based on morphological features and multilocus phylogenetic analyses. The morphological characteristics observed in this study revealed the production of an asexual morph on PDA, with hyphae that were hyaline to light brown, branched, and septate. Conidiophores, which were hyaline and occasionally branched, formed singly or in clusters on the hyphae and measured 15–25.5 × 3.5–6.5 µm. Conidia were single-celled, hyaline, elliptical to oblong, measured 9–14.5 × 4.5–5 µm, with smooth walls and no septa. These morphological features are consistent with those of *C. fructicola* (Wang et al. 2017). Phylogenetic analyses of the combined genes placed the seven new strains within the *C. fructicola* clade, supported by 100% ML, 95% MP, and 1.00 BI, confirming their taxonomic identity within the *Colletotrichum gloeosporioides* species complex (Fig. 5). These findings are consistent with previous records and suggest that this species may be widespread and potentially associated with diverse plant hosts across China.

### ﻿ Colletotrichum siamense

Taxon classificationFungiGlomerellalesGlomerellaceae

Prihast., L. Cai & K.D. Hyde, Fungal Diversity 39: 98 (2009)

93CCC68F-4ECD-5FBD-9F4D-D037906800C3

#### Description.

See Prihastuti et al. (2009), Zhang et al. (2023b), Khuna et al. (2024).

#### Material examined.

China • Yunnan Province, Dali, on *Juglans regia* L., 2023.12.26, Zou Mengting, CXD2-1/GZ18 (dried culture, HGUP 25-0022), living culture GUCC 25-0032; China, • Guizhou Province, Zunyi, Xishui, National Nature Reserve In Xi’an, Pteridophyta, 2024.05.20, coll. Gou Shiqi/Wang Jiaping, Xsj1.1/GZ21 (dried culture, HGUP 25-0023), living culture GUCC 25-0033; • *ibid*. on Pteridophyta, 2024.05.20, coll. Gou Shiqi/Wang Jiaping, xsj1.2/GZ22 (dried culture, HGUP 25-0024), living culture GUCC 25-0034; • *ibid*. on Pteridophyta, 2024.05.20, coll. Gou Shiqi/Wang Jiaping, Xsj1/GZ23 (dried culture, HGUP 25-0025), living culture GUCC 25-0035; China, • Guizhou Province, Tongren, on *Parthenocissus quinquefolia* (L.) Planch., 2024.07.11, coll. Gou Shiqi, D86/FDS40/GZ36 (dried culture, HGUP 25-0034), living culture GUCC 25-0048; • *ibid*. on *Parthenocissus tricuspidata* (Siebold & Zucc.) Planch., 2024.07.11, coll. Gou Shiqi, D93/FDS42/GZ37 (dried culture, HGUP 25-0035), living culture GUCC 25-0049.

#### Notes.

*Colletotrichum siamense*, a member of the *Colletotrichum gloeosporioides* species complex, was described by Weir et al. (2012) and is now recognized as a widespread phytopathogen with a broad host range across multiple countries (Talhinhas and Baroncelli 2021, 2023). It has been reported to cause anthracnose and leaf spot diseases on a variety of economically important crops and ornamental plants (Liu et al. 2017; de Silva et al. 2019; Zhang et al. 2023c). In China, *C. siamense* has been documented on several ornamental and fruit crops, including *Alocasia macrorrhiza* (Huang et al. 2021b), *Viburnum odoratissimum* (Li et al. 2023), *Syzygium samarangense* (Yao et al. 2023), and *Artocarpus heterophyllus* (Khuna et al. 2024). Identification of this species typically relies on morphological characteristics and multilocus phylogenetic analyses, while its pathogenicity is confirmed through inoculation assays following Koch’s postulates. The widespread distribution and extensive host range of *C. siamense* highlight its significance as a potential threat to plant health and agricultural sustainability.

In the present study, strain GUCC 25-0032 isolated from *Camellia japonica*; strains GUCC 25-0033, GUCC 25-0034, and GUCC 25-0035 from unidentified pteridophytes; and strains GUCC 25-0048 and GUCC 25-0049 from *Parthenocissus quinquefolia* and *P. tricuspidata*, respectively, were identified as *C. siamense* based on morphological characteristics, and phylogenetic analyses (Fig. 5). Morphologically, the strains in this study produced an asexual morph on PDA, with hyaline to pale brown, septate, and branched conidiophores. Conidiogenous cells were hyaline to pale brown, cylindrical to ampulliform in shape, and measured 13.4–22.5 × 2.6–4.7 µm. Conidia were single-celled, hyaline, smooth-walled, cylindrical with rounded ends, guttulate, and measured 12.8–19.1 × 4.9–7 µm. Phylogenetic analyses of the combined gene sequences placed the six new strains within the *C. siamense* clade. The species formed a monophyletic clade, supported by a 0.92 BI, with a distinct clade separating it from other species, thereby confirming its taxonomic identity within the *Colletotrichum gloeosporioides* species complex (Fig. 5). This species continues to be reported from diverse host plants in China (He et al. 2011; Zhao et al. 2020; Peng et al. 2022, 2023), and the current study contributes six novel strains to the expanding records of *C. siamense*.

### ﻿ Colletotrichum gloeosporioides

Taxon classificationFungiGlomerellalesGlomerellaceae

(Penz.) Penz. & Sacc., Atti Inst. Veneto Sci. lett., ed Arti, Sér. 6 2(5): 670 (1884)

9225DE64-9122-58B3-9B7A-4A70CAF5923F

#### Description.

See Peng et al. (2023).

#### Material examined.

China • Guizhou Province, Guiyang, Guiyang Botanical Garden Of Medical Plants, symptomatic leaves of *Camellia japonica* L., 2024.4.4, coll. Wang Xingchang, Sch1-1-1/GZ29 (dried culture, HGUP 25-0028), living culture GUCC 25-0041; • *ibid*. on symptomatic leaves of *Camellia japonica* L., 2024.4.4, coll. Wang Xingchang, Sch1-1-4/GZ30 (dried culture, HGUP 25-0029), living culture GUCC 25-0042.

#### Notes.

*Colletotrichum gloeosporioides* is a well-known plant pathogenic species within *Colletotrichum*, responsible for anthracnose diseases across a wide range of host plants (Zhang et al. 2023c). Typical symptoms include dark, sunken lesions on leaves, stems, and fruits, which can significantly impact plant health and yield. This species is of considerable economic importance due to its broad host range, affecting major fruit crops such as papaya, mango, and citrus, as well as ornamental plants like *Camellia japonica* (Peng et al. 2023). Accurate identification of *C. gloeosporioides* relies on a combination of morphological traits, such as the shape and size of conidia and conidiophores, as well as molecular tools, particularly multilocus phylogenetic analyses. Peng et al. (2023) reported the occurrence of *C. gloeosporioides* on *C. japonica*, extending the known host range of the species and underscoring its potential impact on ornamental horticulture.

In the present study, strains GUCC 25-0041 and GUCC 25-0042, isolated from symptomatic tissues of *Camellia japonica*, were identified as *Colletotrichum gloeosporioides* based on both morphological characteristics and multilocus phylogenetic analyses (Fig. 5), consistent with previous findings (Peng et al. 2023). Morphologically, the strains in this study produced an asexual morph on PDA, characterized by hyaline, septate, and branched conidiophores. Conidiogenous cells were cylindrical to ampulliform, measured 12.9–30 × 2.3–3.9 µm, and hyaline. Conidia were single-celled, hyaline, smooth-walled, elliptical to oval with rounded ends, measuring 12.3–16 × 4.9–6.0 µm. Phylogenetic analyses of the combined genes placed the two new strains within the *C. gloeosporioides* species clade. The species clade formed a distinct clade, separated from other species, with support of 75% ML, confirming its taxonomic identity within the *Colletotrichum gloeosporioides* species complex (Fig. 5).

### ﻿ Colletotrichum juglandicola

Taxon classificationFungiGlomerellalesGlomerellaceae

Y. Zhang ter & Lin Zhang bis, MycoKeys 99: 139 (2023)

12AC2A2E-DBE2-5A58-B06D-DBCFACC556D2

Fig. 9

#### Description.

See Zhang et al. (2023a).

#### Material examined.

China • Guizhou Province, Guiyang, Guiyang Botanical Garden Of Medical Plants, *Camellia japonica* L., 2024.4.4, coll. Wang Xingchang, Sch6/GZ31 (dried culture, HGUP 25-0030), living culture GUCC 25-0043; China, • Shandong Province, Taian, Tianwaicun Street, Mountain Tai, *Ilex* L., 2024.02, coll. Wang Xingchang, 2.26-DQ-2/GZ32 (dried culture, HGUP 25-0031), living culture GUCC 25-0044.

#### Notes.

**Figure 9.**
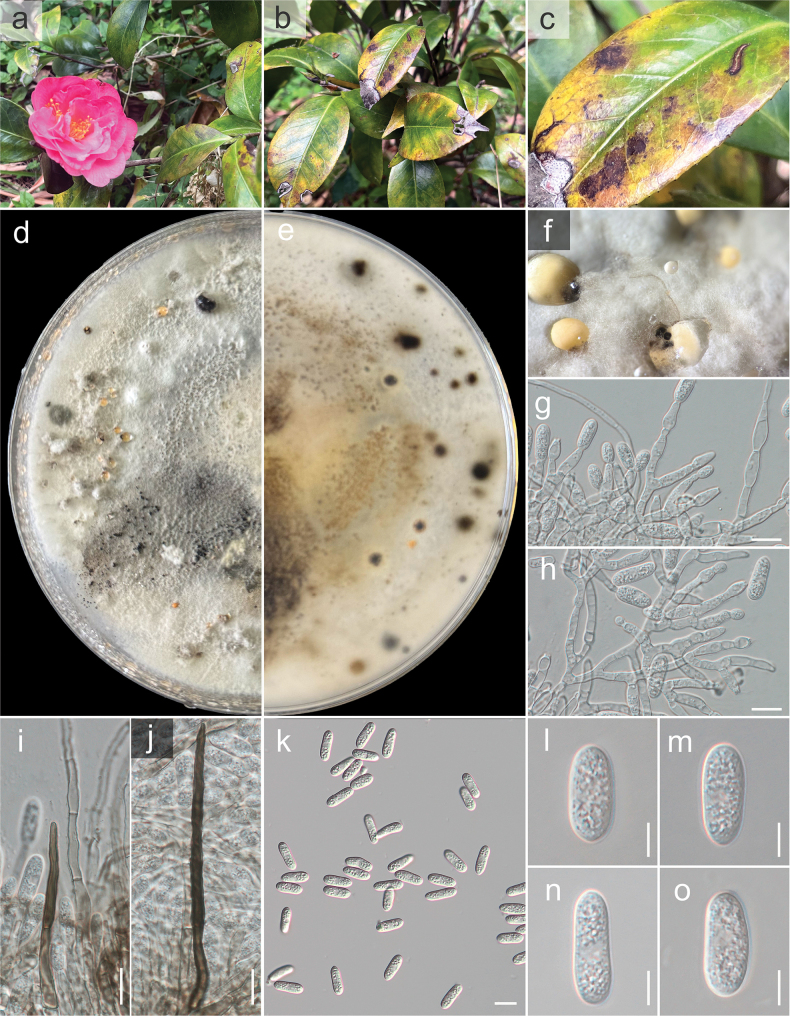
*Colletotrichum juglandicola* (GUCC 25-0043, new host record). a–c. *Camellia japonica*, host habitat; d, e. Culture on OA (d-above, e-reverse; f. Conidiomata on OA; g, h. Conidiophore, conidiogenous cells giving rise to conidia; i, j. Setae; k–o. Conidia. Scale bars: 10 µm (g–k); 5 µm (l–o).

*Colletotrichum juglandicola* was originally described by Zhang et al. (2023a) as the causal agent of anthracnose-like symptoms on *Juglans regia*, characterized by dark, sunken lesions on leaves, stems, and fruit. Zhang et al. (2023a) emphasized the economic impact of *C. juglandicola* in walnut orchards, citing its potential to significantly reduce both yield and nut quality. Like other members of the genus, *C. juglandicola* can infect multiple plant tissues and exhibits a broad host range, making it an important pathogen for integrated disease management. The identification was based on a combination of morphological characteristics, such as the features of conidia and conidiophores and multilocus phylogenetic analyses. In this study, strains GUCC 25-0043 (from *Camellia japonica*) and GUCC 25-0044 (from *Ilex* sp.) were identified as *C. juglandicola* based on both morphological characteristics and multilocus phylogenetic analyses (Figs 5, 9). Morphologically, the strains in this study produced an asexual morph on OA with acervular conidiomata that generated yellow to orange conidial masses; conidiophores were hyaline, smooth-walled, septate, and branched, while setae were medium to dark brown, smooth to finely verruculose near the tip, rounded, 1–3-septate, and 60–110 μm long; conidiogenous cells measured 11–29.5 × 2.7–5 μm, subcylindrical, and either straight or curved. Conidia were hyaline, smooth-walled, subcylindrical, with both ends rounded, measuring 15.8–20.0 × 4.8–6.6 μm, containing 1–3 guttules with granular contents. Phylogenetically, the two new strains grouped with *C. juglandicola* (strain GCMCC 3.2431 and GCMCC 3.24313) with support values of 91% ML and 0.93 PP (Fig. 5). This represents the first report of *C. juglandicola* infecting *C. japonica* and *Ilex* sp. in China, thereby expanding the known host range of the species and highlighting its potential as a pathogen across diverse plant families.

### ﻿ Colletotrichum nanhuaense

Taxon classificationFungiGlomerellalesGlomerellaceae

Z.F. Yu, J. Fungi 8(2, no. 185): 17 (2022)

6FB9C313-240F-56A6-BF0E-B6CA37ABCD62

Fig. 10

#### Description.

See more details in Yu et al. (2022).

#### Material examined.

China • Yunnan Province, Dali, symtomatic fruit of *Juglans regia* L., 2023.12.26, coll. Zou Mengting, CXD7-9/GZ10 (dried culture, HGUP 25-0016), living culture GUCC 25-0026.

#### Notes.

**Figure 10.**
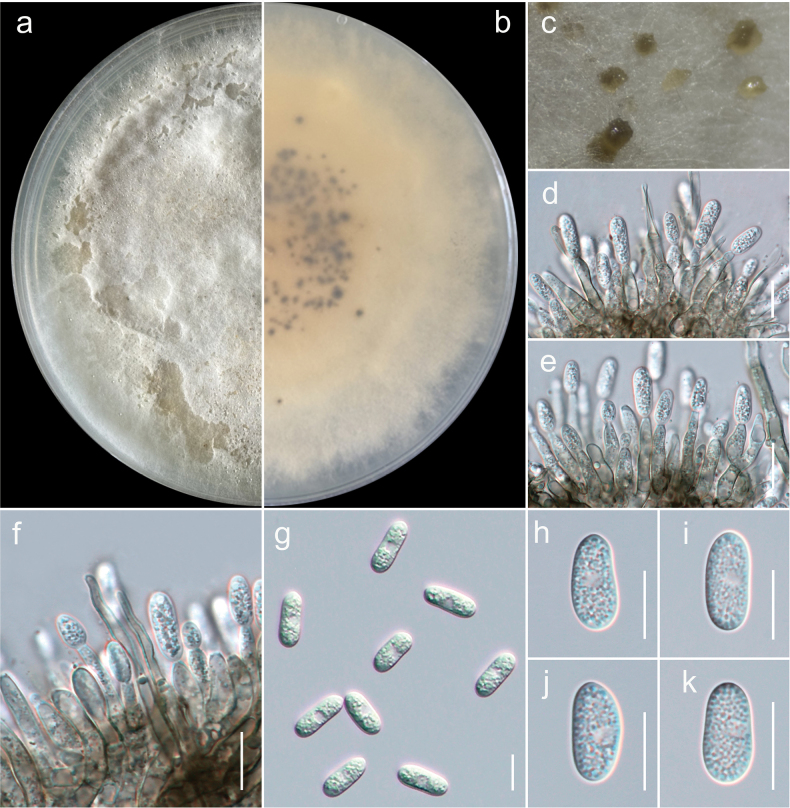
*Colletotrichum nanhuaense* (new host record). a, b. Culture on PDA (a-above, b-reverse); c Conidiomata on PDA; d–f. Conidiophore, conidiogenous cells giving rise to conidia; g–k. Conidia. Scale bars: 10 µm (d–k).

*Colletotrichum nanhuaense* was originally described by Yu et al. (2022) as the causal agent of leaf spot disease on *Ageratina adenophora* in Yunnan Province, China. In the present study, strain GUCC 25-0026, isolated from *Juglans regia*, was identified as *C. nanhuaense* based on both morphological characteristics (Fig. 10) and multilocus phylogenetic analyses (Fig. 5). Morphologically, the strain produced an asexual morph on PDA, with hyaline to dark brown, branched, and septate hyphae. Conidiophores measured 9–29.5 × 3–6.5 µm, were solitary or clustered, hyaline, and unbranched, and were formed on the hyphae. Conidia were single-celled, elliptical to ovoid-shaped, measuring 10.5–15 × 5.5–6 µm, hyaline, with smooth walls and no septa. Phylogenetic analyses placed the new strain within the *C. nanhuaense* clade with strong support (100% ML/1.0 PP; Fig. 5). The detection of *C. nanhuaense* on walnut expands its host range and raises concerns about its impact on other crops. Further research on its biology and spread, especially in *Juglans*, is needed, along with ongoing monitoring. Pathogenicity tests are required to confirm its role in *J. regia*. This is the first report of *C. nanhuaense* on *J. regia* and in China.

### ﻿ Colletotrichum jiangxiense

Taxon classificationFungiGlomerellalesGlomerellaceae

F. Liu & L. Cai, Persoonia 35: 82 (2015)

97BA1826-F2A2-5A53-8466-C13BA2CE40E1

Fig. 12

#### Description.

See Liu et al. (2015), Chang et al. (2024), Zhang et al. (2023b).

#### Material examined.

China, • Yunnan Province, Dali, symptomatic fruit of *Juglans regia* L., 2023.11.12, coll. Zou Mengting, DJ4-6/GZ11 (dried culture, HGUP 25-0017), living culture GUCC 25-0027; • *ibid*. symptomatic fruit of *Juglans regia* L., 2023.11.12, coll. Zou Mengting, DJ11-10/GZ16 (dried culture, HGUP 25-0021), living culture GUCC 25-0031; China, • Guizhou Province, Tongren, on symptomatic leaves of *Pteris henryi* Christ., 2024.07.11, coll. Gou Shiqi, E14/FDS34/GZ40 (dried culture, HGUP 25-0038), living culture GUCC 25-0052; • *ibid*. on symptomatic leaves of *Parthenocissus tricuspidata* (Siebold & Zucc.) Planch., 2024.07.11, coll. Gou Shiqi, E17/FDS41/GZ41 (dried culture, HGUP 25-0039), living culture GUCC 25-0053.

#### Notes.

*Colletotrichum jiangxiense* was initially described as an endophytic fungus associated with *Camellia sinensis* in China (Liu et al. 2015). Since its discovery, the species has been increasingly recognized as a plant pathogen, causing anthracnose and other disease symptoms on various fruit and ornamental hosts. Recent studies have reported its involvement in anthracnose outbreaks in crops such as avocado (Ma et al. 2018; Ayvar Serna et al. 2021; Guo et al. 2022; Zhang et al. 2023b). Additionally, *C. jiangxiense* has been identified as a pathogen in Mexico, where it causes anthracnose on avocado (Ayvar Serna et al. 2021), although it has not yet been reported as a plant pathogen in North America. The international trade of live plants between East Asia and North America raises concerns regarding the potential introduction of *C. jiangxiense* into new environments. Under favorable conditions, this species may shift from an endophytic or low-pathogenic state to a more aggressive pathogenic form, posing a threat to both ornamental and economically important crops. This highlights the importance of phytosanitary surveillance and monitoring for emerging pathogen-host interactions (Liebhold et al. 2012).

**Figure 11.**
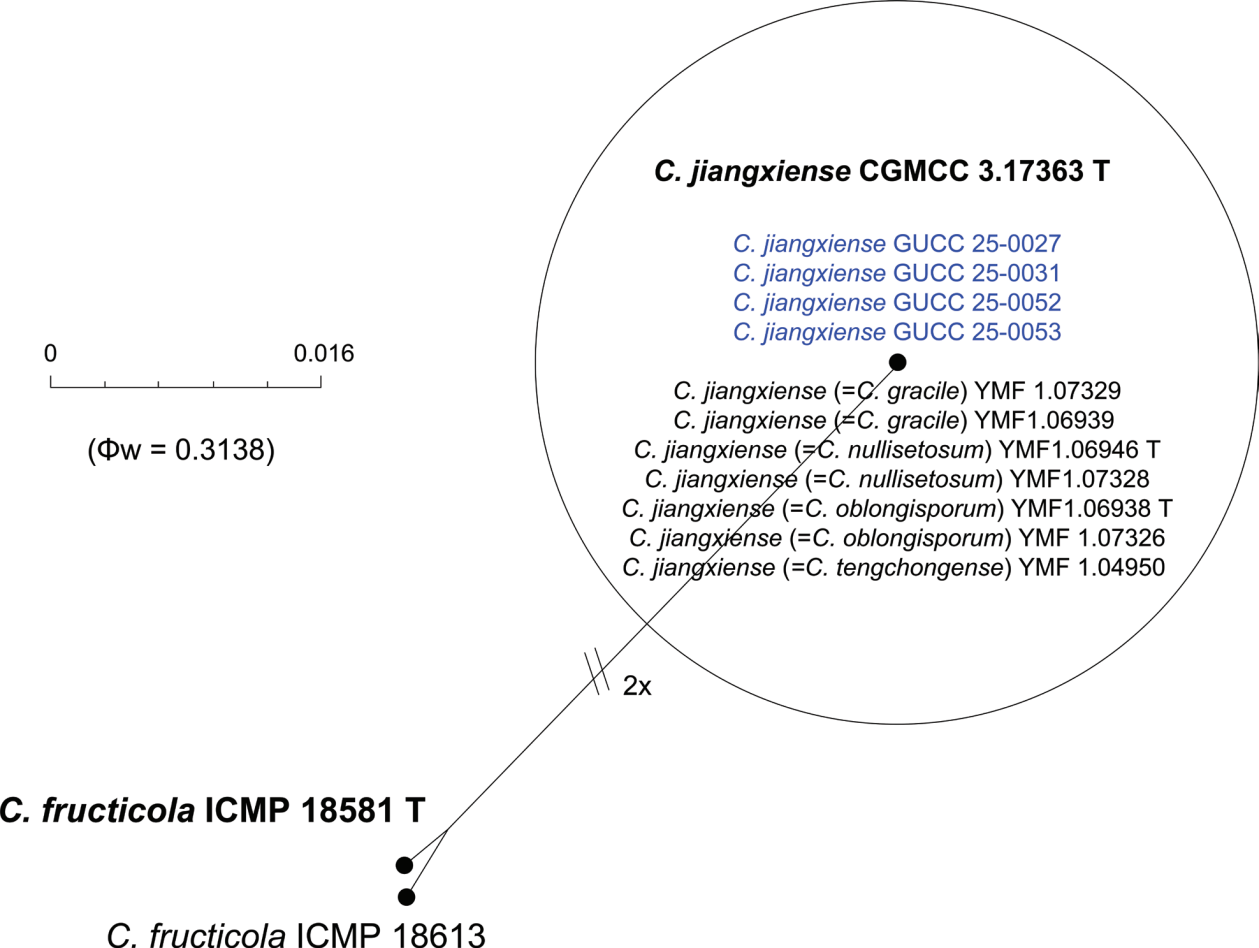
The results of the pairwise homoplasy index (PHI) test for closely related species of *Colletotrichum macroconidii* sp. nov. in this study using both LogDet transformation and splits decomposition. PHI test results (Φw) > 0.05 indicate no significant recombination within the dataset.

*Colletotrichum jiangxiense* is identified based on both its morphological characteristics and phylogenetic placement, supported by multilocus gene sequence data (Liu et al. 2015). In this study, we report the first identification of *C. jiangxiense* on *Juglans regia*, *Pteris henryi*, and *Parthenocissus tricuspidata*, thus expanding its known host range. Strains GUCC 25-0027 and GUCC 25-0031, isolated from *J. regia*, and strains GUCC 25-0052 and GUCC 25-0053, isolated from *P. henryi* and *P. tricuspidata*, respectively, were identified as *C. jiangxiense* through morphological and multilocus phylogenetic analyses (Figs 5, 11). Phylogenetically, four strains clustered with *C. jiangxiense* in both combined gene tree and single loci tree, showing high sequence similarity and only minor genetic differences from the type strain (CBS 515.78): GUCC 25-0027 (ITS = 100% [0/514], *gapdh* = 100% [0/252], *chs-1* = 100% [0/287], *act* = 100% [0/273], *β-tubulin* = 100% [0/689], *cal* = 100% [0/733], *ApMat* = 99% [2/949]); GUCC 25-0031 (ITS = 100% [0/514], *gapdh* = 100% [0/252], *chs-1* = 100% [0/287], *act* = 100% [0/273], *β-tubulin* = 100% [0/700], *cal* = 100% [0/757], *ApMat* = 100% [0/950]); GUCC 25-0052 (ITS = 100% [0/514], *gapdh* = 100% [0/252], *chs-1* = 100% [0/287], *act* = 100% [0/273], *β-tubulin* = 100% [0/732], *cal* = n/a, *ApMat* = n/a); and GUCC 25-0053 (ITS = 100% [0/514], *gapdh* = 100% [0/252], *chs-1* = 100% [0/287], *act* = 100% [0/273], *β-tubulin* = 100% [0/732], *cal* = 100% [0/751], *ApMat* = 99% [1/944]). These results indicate only minor genetic variation from *C. jiangxiense*. This placement was further supported by the PHI test, which indicated significant recombination among the two collections (Fig. 11). Morphologically, the strains exhibit hyaline, smooth-walled, aseptate, and unbranched conidiophores that grow directly from the hyphae. Conidiogenous cells were hyaline, cylindrical to clavate, measuring 12.6–27.5 × 3.2–4.6 µm, smooth-walled, and lacked a collarette. Conidia were cylindrical, with both ends bluntly rounded, or one end bluntly rounded and the other acutely rounded, measuring 14.6–18 × 4.9–6.5 µm, aseptate, and smooth-walled. Appressoria were brown to pale-brown, circular, subglobose to ellipsoidal or irregular, with lobed margins, measuring 5–9 × 5–8 µm. These morphological characteristics are consistent with those of *C. jiangxiense* as described by Liu et al. (2015).

**Figure 12.**
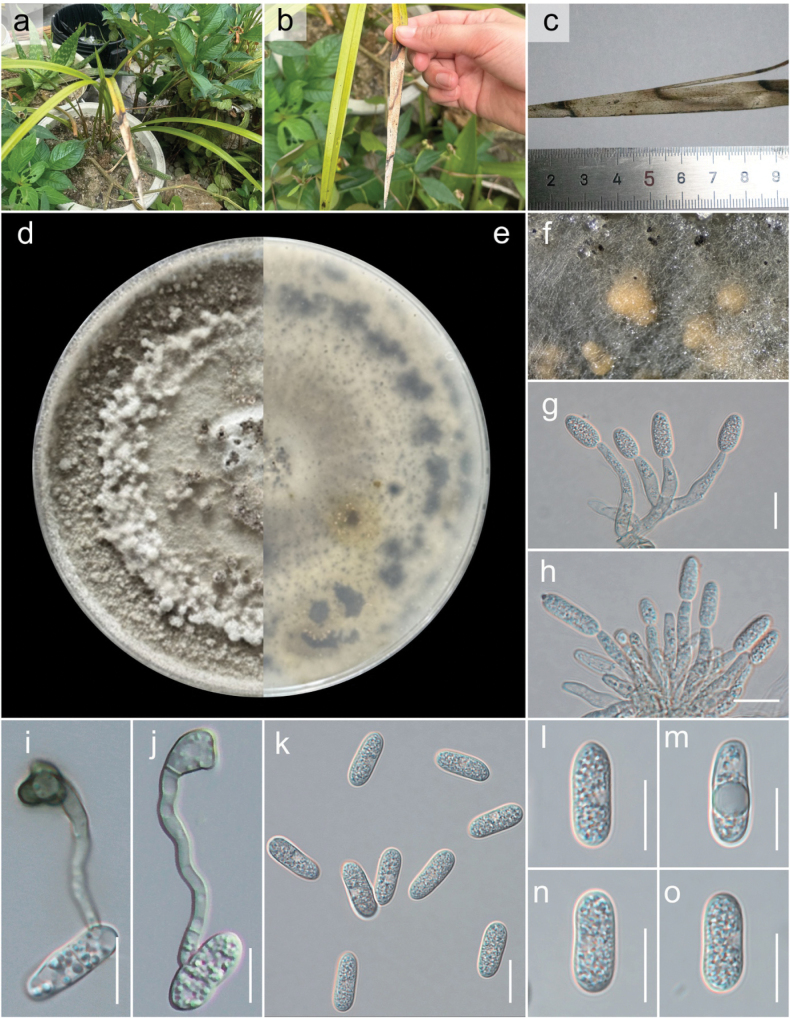
*Colletotrichum jiangxiense* (new host record). a–c. *Pteris henryi*, host habitat; d, e. Culture on OA (d-above, e-reverse); f. Conidiomata on OA; g, h. Conidiophores, conidiogenous cells giving rise to conidia; i, j. Appressoria; k–o. Conidia. Scale bars: 10 µm (i, j); 5 µm (g, h, k–o).

Phylogenetic analysis placed the four new strains within a distinct clade, which also includes *C. nullisetosum*, *C. oblongisporum*, *C. gracile*, and *C. tengchongense*. This clade is strongly supported by 98% ML and 0.99 PP values (Fig. 5). However, *C. nullisetosum*, *C. oblongisporum*, and *C. gracile* are currently considered as nomenclaturally invalid (nom. inval.) in MycoBank, due to violations of Article 40.8 of the International Code of Nomenclature. Based on their morphological characteristics (Yu et al. 2022) and taxonomic status, we treat these three species as synonyms of *C. jiangxiense*. *Colletotrichum tengchongense* is retained as a valid species name within the clade. Morphologically, it differs from *C. jiangxiense* by the structure of its conidiophores and conidiogenous cells, with *C. tengchongense* producing lageniform or ampulliform conidiogenous cells (Liu et al. 2015; Zheng et al. 2022). Phylogenetically, *C. tengchongense* forms a relatively long branch within the *C. jiangxiense* species clade. However, its sequence similarity with the type strain of *C. jiangxiense* indicates only minor genetic differences: ITS = 0% [0/515], *gapdh* = 0% [0/274], *chs-1* = 0.3% [1/287], *act* = 0.4% [1/246], *β-tubulin* = 0% [0/700], *cal* = 0.1% [1/715], *ApMat* = 0.2% [2/870]. These results suggest very limited genetic variation *from C. jiangxiense*, and we therefore treat *C. tengchongense* as conspecific with *C. jiangxiense*. This conclusion was further supported by the PHI test, which indicated significant recombination within the *C. jiangxiense* species group (Fig. 11). Nevertheless, further studies incorporating additional loci are needed to confirm the phylogenetic placement of this taxon.

### ﻿ Colletotrichum magnum

Taxon classificationFungiGlomerellalesGlomerellaceae

(S.F. Jenkins & Winstead) Rossman & W.C. Allen, IMA Fungus 7(1): 4 (2016)

97416470-0664-5FDC-9B8E-2D786B4811D2

Fig. 14

#### Description.

See Rossman et al. (2016) and Damm et al. (2019).

#### Material examined.

China • Yunnan Province, Dali, symptomatic fruit of *Juglans regia* L., 2023.11.5, coll. Zou Mengting, RH1-5/GZ13 (dried culture, HGUP 25-0019), living culture GUCC 25-0029; • *ibid*. symptomatic fruit of *Juglans regia* L., 2023.11.12, coll. Zou Mengting, DJ5-8/GZ15 (dried culture, HGUP 25-0020), living culture GUCC 25-0030.

#### Notes.

*Colletotrichum magnum* is a well-recognized pathogen responsible for anthracnose disease across a wide range of plant hosts, including both agricultural crops and ornamental species, causing considerable economic losses (Rossman et al. 2016; Damm et al. 2019). The species was first described as distinct based on its unique morphological characteristics and phylogenetic placement (Rossman et al. 2016). Key diagnostic features for identifying *C. magnum* include its cylindrical conidia and the structure of its cylindrical to ellipsoidal conidiogenous cells, supported by molecular phylogenetic analyses using multiple genes (Rossman et al. 2016; Damm et al. 2019).

**Figure 13.**
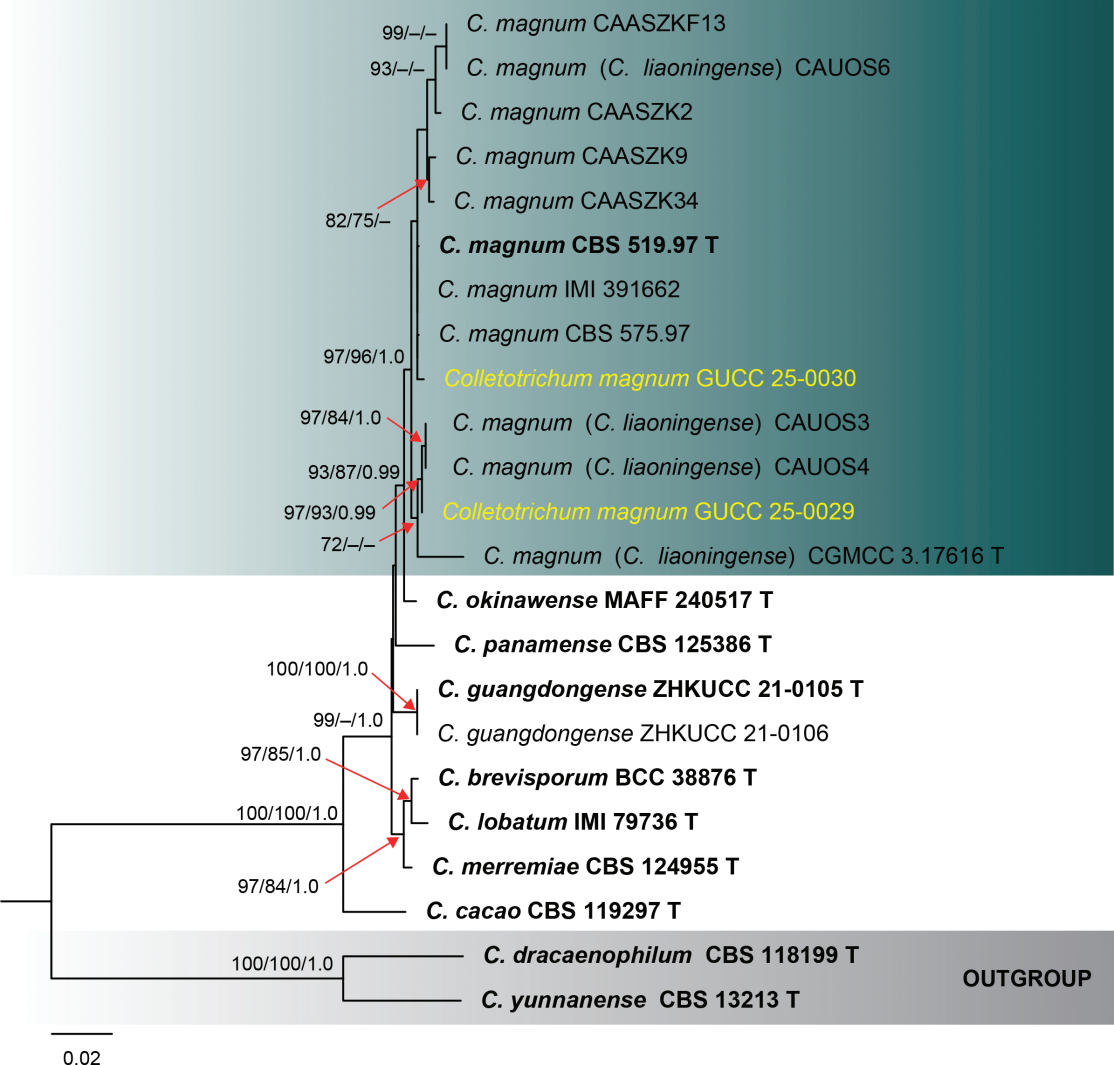
Phylogenetic tree constructed using a maximum likelihood (ML) analysis based on a combined ITS, *gapdh*, *chs-1*, *act*, *β-tubulin*, and *his3* sequences, representing *Colletotrichum magnum* species complex. The tree topology of the ML analysis was identical to the Maximum Parsimony (MP) and Bayesian posterior probability (BI) analysis. The final RAxML tree with a likelihood value of -6752.888091is presented here. The evolutionary model GTR+GAMMA was applied to all the genes. The analysis included twenty-three (23) taxa with a total of 2449 characters, with 434 distinct alignment patterns, and 16.59% were gaps and undetermined characters. Estimated base frequencies were as follows: A = 0.221259, C = 0.309082, G = 0.253858, T = 0.215801; substitution rates AC = 0.914360, AG = 2.159987, AT = 0.604764, CG = 0.664359, CT = 4.006750, GT = 1.0; gamma distribution shape parameter *α* = 0.280788; tree length = 0.381194. Bootstrap support values for ML and MP ≥ 70% and Bayesian Posterior Probabilities (BI) ≥ 0.90 are indicated at the nodes as ML/MP/BI. The tree is rooted with *C. dracaenophilum* (CBS 118199) and *C. yunnanense* (CBS 13213). Type strains are denoted in bold and T and sequences generated in this study are in yellow. Bar = 0.02 represents the estimated number of nucleotide substitutions of site per branch.

**Figure 14.**
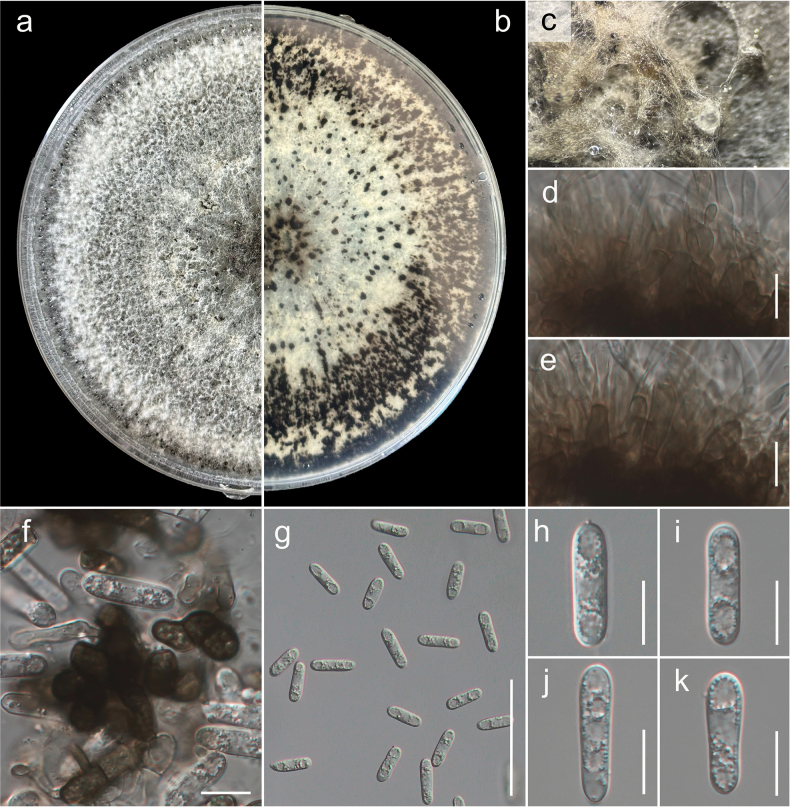
*Colletotrichum magnum* (GUCC 25-0029, new record). a, b. Culture on OA (a-above, b-reverse); c. Conidiomata on OA; d, e. Conidiophore, conidiogenous cells giving rise to conidia; f. Chlamydospores; g–k. Conidia. Scale bars: 10 µm (d–k).

In China, *Colletotrichum* species have been commonly associated with *Juglans regia* (English walnut), causing leaf spots and lesions that reduce photosynthetic capacity and ultimately impact yield (Zhu et al. 2014; Wang et al. 2017; He et al. 2019; Li et al. 2023, 2024; Zhang et al. 2023b). Although the full pathogenic potential of *C. magnum* remains under investigation, it is increasingly regarded as an emerging threat to walnut production, especially in regions where walnuts are economically important. In the present study, strains GUCC 25-0029 and GUCC 25-0030, isolated from symptomatic fruit of *J. regia*, were identified as *Colletotrichum magnum* based on morphological characteristics (Fig. 14) and multilocus phylogenetic analyses (Fig. 13). Morphologically, the strain produced an asexual morph on OA, with transparent to dark brown, branched, and septate hyphae. Conidiogenous cells were cylindrical to ellipsoidal, measured 25.5–30 × 3.5–4.5 µm, solitary or clustered, hyaline, and unbranched, formed on the hyphae. Conidia were hyaline, cylindrical, straight, the apex and base rounded, measured 7.5–17.5 × 2.5–4.5 µm, hyaline, with smooth walls and no septa (Fig. 14). Phylogenetic analyses placed the new strain within the *C. magnum* clade with support, 97% ML, 96% MP, and 1.0 PP (Fig. 13). This represents the first report of *C. magnum* infecting *Juglans regia* in China. However, to confirm its pathogenic role on *J. regia*, future pathogenicity tests are required.

## ﻿Discussion

Identification of *Colletotrichum* species is vital for understanding their biology, host range, pathogenic potential, and for developing effective disease management strategies. Although morphological traits such as conidial shape, size, and colony appearance have traditionally been used for species delimitation, these features often overlap among taxa, especially within species complexes, making morphology-based identification unreliable (Damm et al. 2012a, b; Cannon et al. 2012). Molecular data have become essential for resolving taxonomic ambiguities in *Colletotrichum* (Marin-Felix et al. 2017; Bhunjun et al. 2021; Jayawardena et al. 2021; Liu et al. 2022). Multilocus phylogenetic analyses using markers such as ITS, *gapdh*, *act*, *chs-1*, *β-tubulin*, *cal*, *his3*, and *ApMat* have significantly improved species resolution, especially within the *C. gloeosporioides*, *C. acutatum*, and *C. boninense* complexes (Weir et al. 2012; Jayawardena et al. 2021; Liu et al. 2022). These methods are crucial for distinguishing cryptic species, correcting misidentifications, and resolving invalid taxa, particularly in a genus exhibiting high morphological plasticity.

In the present study, our phylogenetic analyses confirmed that four newly collected strains clustered within a robust clade that includes *Colletotrichum jiangxiense*, *C. nullisetosum*, *C. oblongisporum*, *C. gracile*, and *C. tengchongense*. These findings align with earlier reports by Liu et al. (2015) and Yu et al. (2022), who highlighted morphological similarity and close phylogenetic placement among these taxa. However, *C. nullisetosum*, *C. oblongisporum* and *C. gracile* are now considered nomenclaturally invalid (nom. inval.) due to violations of the International Code of Nomenclature, and based on our morphological and molecular data, we support their synonymy under *C. jiangxiense*. This clarification builds on previous taxonomic suggestions and provides new evidence from fresh collections in Guizhou and Yunnan. *Colletotrichum tengchongense*, described by Zheng et al. (2022), was initially recognized as a distinct taxon due to its lageniform to ampulliform conidiogenous cells and subtle colony differences. In our analyses, however, *C. tengchongense* shows only minimal nucleotide differences when compared with *C. jiangxiense* (ITS = 0% [0/515], *gapdh* = 0% [0/274], *chs-1* = 0.3% [1/287], *act* = 0.4% [1/246], *β-tubulin* = 0% [0/700], *cal* = 0.1% [1/715], *ApMat* = 0.2% [2/870]). These differences fall well within the range of intraspecific variation commonly reported for *Colletotrichum* species complexes (Cannon et al. 2012; Hyde et al. 2014; Jayawardena et al. 2021). Moreover, phylogenetic analyses consistently recover *C. tengchongense* and *C. jiangxiense* as a monophyletic lineage, without evidence of independent sub-clade formation or unique lineage divergence. Similar cases of low genetic divergence but close phylogenetic clustering have previously been used to synonymize taxa in *Colletotrichum* (Damm et al. 2012a, b; Liu et al. 2022). Taken together, both sequence identity and phylogenetic topology provide strong evidence that *C. tengchongense* does not represent a distinct species and should be treated as a later synonym of *C. jiangxiense*. Our data also support the synonymization of *C. speciosum* and *C. simulanticitri* with *C. nymphaeae*, corroborating the conclusions of Yu et al. (2022). Both taxa clustered tightly with *C. nymphaeae*, showing negligible sequence divergence. *Colletotrichum simulanticitri*, although based on four loci, exhibited low genetic distances and evidence of recombination, justifying its synonymy. Meanwhile, *C. speciosum*, described from ITS data only, lacked the molecular support necessary for species distinction. Both names are also currently listed as nom. inval. due to violations of the International Code of Nomenclature. They are considered synonyms here based on molecular and taxonomic criteria. This finding strengthens earlier assumptions based on partial gene data and provides a more comprehensive resolution using a broader phylogenetic framework. Altogether, the integration of our current results with previous taxonomic and phylogenetic studies highlights the crucial role of molecular data in resolving species boundaries in *Colletotrichum*. Given the high morphological plasticity and broad host associations across the genus, molecular tools remain essential for accurate species identification and understanding species complexes (Liu et al. 2017; de Silva et al. 2019; Peng et al. 2023; Zhang et al. 2023a, b, c; Trkulja et al. 2024; Lu et al. 2025).

This study significantly contributes to our understanding of *Colletotrichum* diversity in southwestern China by presenting a novel species, *Colletotrichum macroconidii*, alongside 11 previously known species, including six new host and regional records. The integration of detailed morphological analyses with multilocus phylogenetic data allowed us to clarify the taxonomic relationships of these species, offering valuable insights into their ecological and pathogenic roles in southwestern China (Hyde et al. 2024; Damm et al. 2009).

Our phylogenetic analyses revealed support for the distinctiveness of the newly described species, *C. macroconidii*, which was consistently placed as a sister clade to *C. gardeniae* within the *C. gloeosporioides* species complex. This novel taxon is characterized by its large, aseptate conidia, a feature that differentiates it from closely related species within the same complex. The phylogenetic distinctiveness of *C. macroconidii* was further supported by significant genetic variations, along with the absence of recombination between *C. macroconidii* and its closest relative, as demonstrated by the Genealogical Concordance Phylogenetic Species Recognition (GCPSR) model and the pairwise homoplasy index (Φw) test (Quaedvlieg et al. 2014; Damm et al. 2019). These results provide robust molecular evidence for recognizing *C. macroconidii* as a separate species within the *C. gloeosporioides* complex.

Additionally, we report six new host or geographic records for China: *C. fioriniae*, *C. trichellum*, *C. juglandicola*, *C. nanhuaense*, *C. jiangxiense*, and *C. magnum*. Notably, *C. trichellum* is reported for the first time from *Hedera* spp. in China, marking an important expansion of its known host range. *Colletotrichum trichellum* has been previously reported from various countries, including Canada, Germany, and the United Kingdom, where it causes leaf and stem spots on *Hedera helix* (English ivy) (Jayawardena et al. 2021). Our finding extends the host range of *C. trichellum* and highlights its potential to impact ornamental plants in China. This is particularly concerning as *Hedera helix* is widely used in landscaping, and the pathogen could have significant economic and ecological effects (Jayawardena et al. 2021). Similarly, *C. juglandicola*, first described from *Juglans regia* (walnut) in China, was detected in new host plants, including *Camellia japonica* and *Ilex* spp. These findings underscore the broad host range of *C. juglandicola* and its emerging importance as a pathogen of ornamental species in China (Zhang et al. 2023a). Additionally, *C. magnum*, a known anthracnose pathogen (Rossman et al. 2016; Damm et al. 2019), was identified from *Juglans regia* for the first time in China. Given the importance of walnut cultivation in the region, this represents a significant addition to the list of *Colletotrichum* species affecting economically valuable crops (Rossman et al. 2016).

New host associations were also confirmed for *C. fioriniae*, *C. nanhuaense*, and *C. jiangxiense*, which were identified on new hosts, *Parthenocissus tricuspidata* and *Actinidia chinensis* (kiwifruit). These findings further emphasize the ecological adaptability of *Colletotrichum* species and their ability to infect diverse plant species across different regions. In particular, *C. fioriniae*, traditionally associated with fruits like pear, has now been recorded infecting *P. tricuspidata*, highlighting the need for continued surveillance of its host range (Yu et al. 2022; Wang et al. 2025).

In conclusion, our integrative approach, combining multilocus phylogenetic analyses with morphological evidence, not only confirms species identities but also clarifies synonymy and reveals new host-pathogen associations (Dean et al. 2012; Jayawardena et al. 2020). This study reinforces the essential role of molecular phylogenetics in modern fungal taxonomy and expands the known diversity of *Colletotrichum* in China, with implications for agriculture, forestry, and biodiversity conservation.

## Supplementary Material

XML Treatment for Colletotrichum fioriniae

XML Treatment for Colletotrichum nymphaeae

XML Treatment for Colletotrichum metake

XML Treatment for Colletotrichum trichellum

XML Treatment for Colletotrichum macroconidii

XML Treatment for Colletotrichum fructicola

XML Treatment for Colletotrichum siamense

XML Treatment for Colletotrichum gloeosporioides

XML Treatment for Colletotrichum juglandicola

XML Treatment for Colletotrichum nanhuaense

XML Treatment for Colletotrichum jiangxiense

XML Treatment for Colletotrichum magnum

## ﻿Additional information

### Use of AI

No use of AI was reported.

### Author ORCIDs

Chada Norphanphoun https://orcid.org/0000-0002-5756-7206

Meng-Ting Zou https://orcid.org/0009-0005-5564-4131

Yong Wang https://orcid.org/0000-0003-3831-2117

### Data availability

All of the data that support the findings of this study are available in the main text.
